# The effect of ageing and osteoarthritis on the mechanical properties of cartilage and bone in the human knee joint

**DOI:** 10.1038/s41598-018-24258-6

**Published:** 2018-04-12

**Authors:** Abby E. Peters, Riaz Akhtar, Eithne J. Comerford, Karl T. Bates

**Affiliations:** 1 Department of Musculoskeletal Biology, Institute of Ageing and Chronic Disease, University of Liverpool, The William Henry Duncan Building, 6 West Derby Street, Liverpool, L7 8TX UK; 2 Department of Mechanical, Materials and Aerospace Engineering, School of Engineering, University of Liverpool, The Quadrangle, Brownlow Hill, Liverpool, L69 3GH UK; 3 Institute of Veterinary Science, Leahurst Campus, University of Liverpool, Chester High Road, Neston, Wirral, CH64 7TE UK

## Abstract

Osteoarthritis is traditionally associated with cartilage degeneration although is now widely accepted as a whole-joint disease affecting the entire osteochondral unit; however site-specific cartilage and bone material properties during healthy ageing and disease are absent limiting our understanding. Cadaveric specimens (n = 12; 31–88 years) with grades 0–4 osteoarthritis, were dissected and spatially correlated cartilage, subchondral and trabecular bone samples (n = 8 per cadaver) were harvested from femoral and tibial localities. Nanoindentation was utilised to obtain cartilage shear modulus (*G*′) and bone elastic modulus (*E*). Cartilage *G*′ is strongly correlated to age (*p* = 0.003) and osteoarthritis grade (*p* = 0.007). Subchondral bone *E* is moderately correlated to age (*p* = 0.072) and strongly correlated to osteoarthritis grade (*p* = 0.013). Trabecular bone *E* showed no correlation to age (*p* = 0.372) or osteoarthritis grade (*p* = 0.778). Changes to cartilage *G*′ was significantly correlated to changes in subchondral bone *E* (*p* = 0.007). Results showed preferential medial osteoarthritis development and moderate correlations between cartilage *G*′ and sample location (*p* = 0.083). Also demonstrated for the first time was significant correlations between site-matched cartilage and subchondral bone material property changes during progressive ageing and osteoarthritis, supporting the role of bone in disease initiation and progression. This clinically relevant data indicates a causative link with osteoarthritis and medial habitual loading.

## Introduction

Osteoarthritis (OA) is one of the most prevalent musculoskeletal conditions amongst the adult population with the most common diagnosis at the knee joint^1^. Individuals with OA have increased variability in gait spatial-temporal parameters^2^, which in turn can lead to a decline in locomotor stability and increase the risk of falls through reduced functionality^3^. Ageing is a high risk factor for the development and progression of knee OA and is known to influence mechanical and biochemical changes within tissue structure, even in the absence of OA and other disease or injury status^4,5^.

OA is traditionally associated with degeneration of the articular cartilage; however it is now more widely accepted that OA is a whole-joint disease that alters the integrity of multiple tissues of the osteochondral unit^6^. A recent review suggests tissue-level adaptations of the subchondral bone are thought to occur in OA prior to degeneration of the overlying articular cartilage;^7^ however this has been rarely explored in the human knee joint. Abnormal remodeling of the subchondral bone has been identified, including high proliferation of volume at the bone-cartilage interface, with observations of changes to density, separation and quantity of the trabecular bone^8,9^. These structural modifications of bone and cartilage occur in synergy further suggesting subchondral bone plays an important but mostly unexplored role in the initiation and progression of the disease^10^.

These structural adaptations may logically influence the mechanical strength of such tissues. Research shows that cartilage elastic modulus (*E*) declines with progressive grades of OA^11,12^. However, there is minimal research on the effect of OA on subchondral bone material properties. Indeed there has been no comparison of the material properties of site matched cartilage and bone from the same donor in the presence of OA when compared to healthy controls. Knowledge of material properties of all tissues involved would enhance the development of treatment and clinical outcomes by advancing our understanding of disease mechanisms^13^.

Subsequently the aim of this research is to systematically quantify age and OA related trends in the material properties of multiple tissues from the human knee joint. Articular cartilage, subchondral bone and trabecular bone samples from a cohort of donors spanning a large age range are tested using nanoindentation techniques. This study includes samples with varying grades of OA in order to understand how ageing and disease status affects multiple tissues of the knee joint simultaneously. Extraction of multiple, spatially distributed samples of all tissues from the same donors allows us to explicitly test for localised changes within tissues and furthermore for correlated changes between tissues during ageing and OA progression for the first time.

## Results

Overall cartilage shear storage modulus (*G*′) (0.14–1.30 MPa), subchondral bone *E* (11.12–15.33 GPa) and trabecular bone *E* (10.75–13.66 GPa) varied considerably across cadavers. The average mean and standard deviation (SD) across samples from the whole joint for all tissues can be seen in Table 1, along with age and grade of OA of the cadaver. Note that results herein present cartilage *G*′ and subchondral and trabecular bone *E*. Values of all parameters including the addition of bone hardness (*H*), cartilage shear loss modulus (*G*″) and cartilage loss factor can be found in Supplementary Material 1.

**Table 1:** Mean and standard deviation (SD) of cartilage shear storage modulus (*G*′) (MPa), subchondral bone elastic modulus (*E*) (GPa) and trabecular bone elastic modulus (*E*) (GPa) for samples across the whole joint.

	Age (Years)	Gender	Limb	OA ICRS Grade*	Cartilage *G′* (MPa)	Subchondral Bone *E* (GPa)	Trabecular Bone *E* (GPa)
					Mean ± SD	Mean ± SD	Mean ± SD
Cadaver 1	31	Female	Left	Grade 0	1.30 ± 0.65	—	13.13 ± 3.34
Cadaver 2	37	Female	Left	Grade 0	1.0 ± 0.74	11.96 ± 1.90	12.29 ± 2.87
Cadaver 3	43	Female	Right	Grade 0	0.90 ± 0.55	11.89 ± 1.64	11.67 ± 2.88
Cadaver 4	49	Male	Left	Grade 0–1	0.65 ± 0.51	—	13.37 ± 2.16
Cadaver 5	51	Male	Right	Grade 0–1	0.96 ± 0.50	12.83 ± 1.64	13.09 ± 2.75
Cadaver 6	58	Male	Right	Grade 1–2	0.41 ± 0.54	11.12 ± 2.18	10.75 ± 2.90
Cadaver 7	72	Male	Right	Grade 2–3	0.14 ± 0.31	14.18 ± 1.99	12.13 ± 3.78
Cadaver 8	72	Male	Left	Grade 1–3	0.55 ± 0.45	14.34 ± 2.03	13.66 ± 3.13
Cadaver 9	79	Male	Left	Grade 1–2	0.15 ± 0.09	14.31 ± 1.57	12.29 ± 3.89
Cadaver 10	80	Male	Left	Grade 1–4	0.31 ± 0.48	15.33 ± 1.70	12.08 ± 2.68
Cadaver 11	86	Female	Right	Grade 0–1	0.40 ± 0.34	13.76 ± 1.93	11.64 ± 3.21
Cadaver 12	88	Male	Right	Grade 1–3	0.27 ± 0.36	14.30 ± 1.68	12.43 ± 2.63

Age, osteoarthritis (OA) International Cartilage Repair Society (ICRS) grade (0–4) and limb side is also shown. *Note. OA grade is based on all 8 samples extracted, hence multiple grades per cadaver due to regional spatial variation in OA across the joint.

### Effect of Ageing

**Figure 1:**
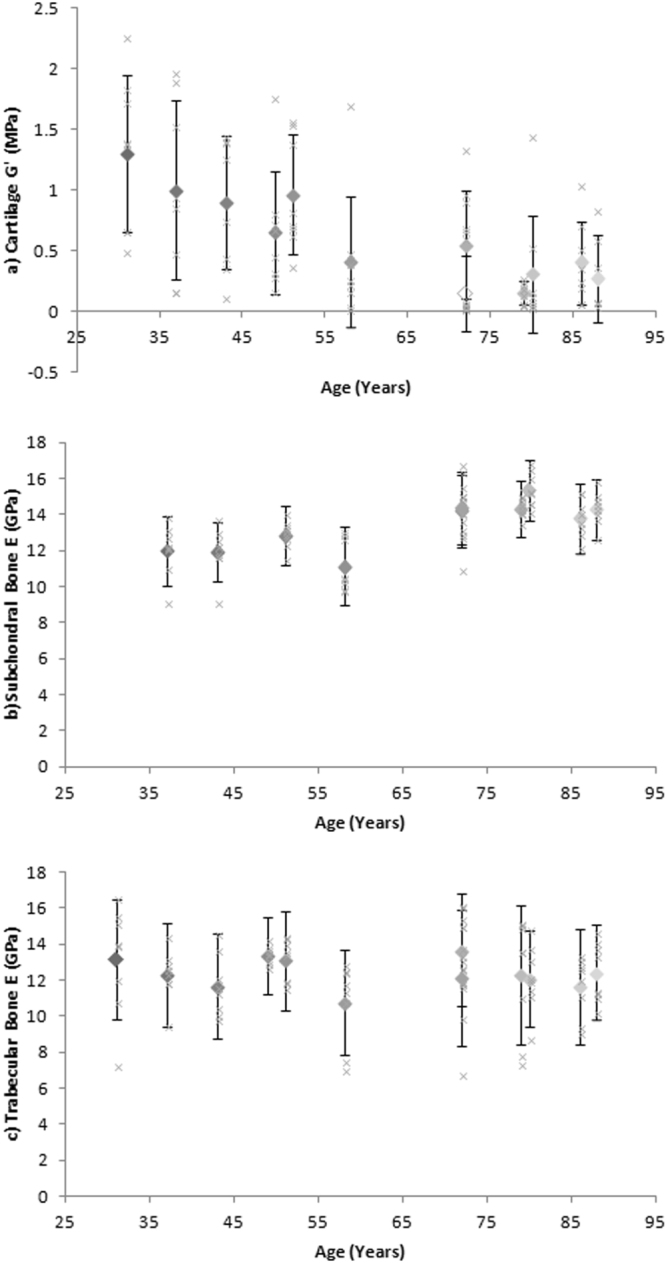
Mean of combined sample (**a**) Cartilage shear storage modulus (*G*′) (MPa), (**b**) Subchondral bone elastic modulus (*E*) (GPa) and (**c**) Trabecular bone elastic modulus (*E*) (GPa) correlated to age (diamonds). Error bars represent standard deviation (SD). The mean of each eight individual spatially correlated samples from cadavers correlated against age (crosses).

Increasing age is strongly correlated to a decrease in cartilage *G*′ (*τ*b = −0.657, *p* = 0.003) and moderately correlated to an increase in subchondral bone *E* (*τ*b = 0.449, *p* = 0.072) using overall joint means. However there is no significant correlation between increasing age and trabecular *E* (*τ*b = −0.198; *p* = 0.372).These trends are shown in Figure 1 by combined sample mean and SD plotted against age, along with the mean of each of the eight individual spatially correlated samples.

Increasing age was also strongly correlated to cartilage *G*′′ (*τ*b = −0.565; *p* = 0.011) and cartilage *E* (*τ*b = −0.657; *p* = 0.003), and moderately correlated to cartilage loss factor (*τ*b = −0.462; *p* = 0.039), subchondral bone *H* (*τ*b = 0.276; *p* = 0.277) and trabecular bone *H* (*τ*b = 0.394; *p* = 0.083) (calculated using Kendall’s Tau-b for overall joint means) (see values in Supplementary Material 1).

### Effect of Osteoarthritis

**Figure 2:**
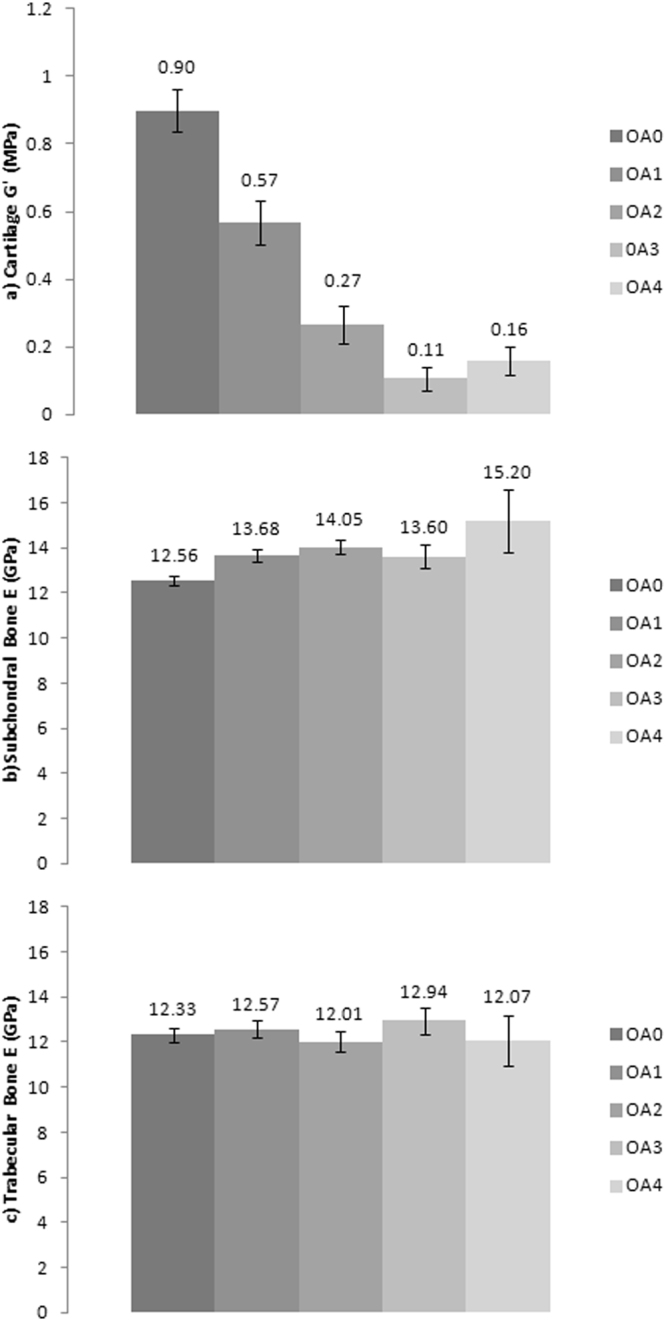
The relationship between (**a**) Cartilage shear storage modulus (*G*′) (MPa), (**b**) Subchondral bone elastic modulus (*E*) (GPa), and (**c**) Trabecular bone elastic modulus (*E*) (GPa) to osteoarthritis International Cartilage Repair Society (ICRS) grade (0–4). Error bars represent 95% confidence interval and figures represent standard deviation (SD).

Increasing grade of OA is correlated to a decrease in cartilage *G*′ (*τ*b = −0.625; *p* = 0.007) and an increase in subchondral bone *E* (*τ*b = 0.645; *p* = 0.013) using overall joint grading (Fig. 2). Trabecular bone *E* showed no significant correlation between overall joint OA grade (*τ*b = −0.066; *p* = 0.778) (Fig. 2).

Overall joint grade of OA was strongly correlated to cartilage *G*′′ (*p* = 0.002), cartilage loss factor (*p* = 0.006), cartilage *E* (*p* = 0.007) and subchondral bone *H* (*p* = 0.033), and moderately correlated to trabecular bone *E* (*p* = 0.087) (calculated using Kendall’s Tau-b). (see values in Supplementary Material 1).

**Figure 3:**
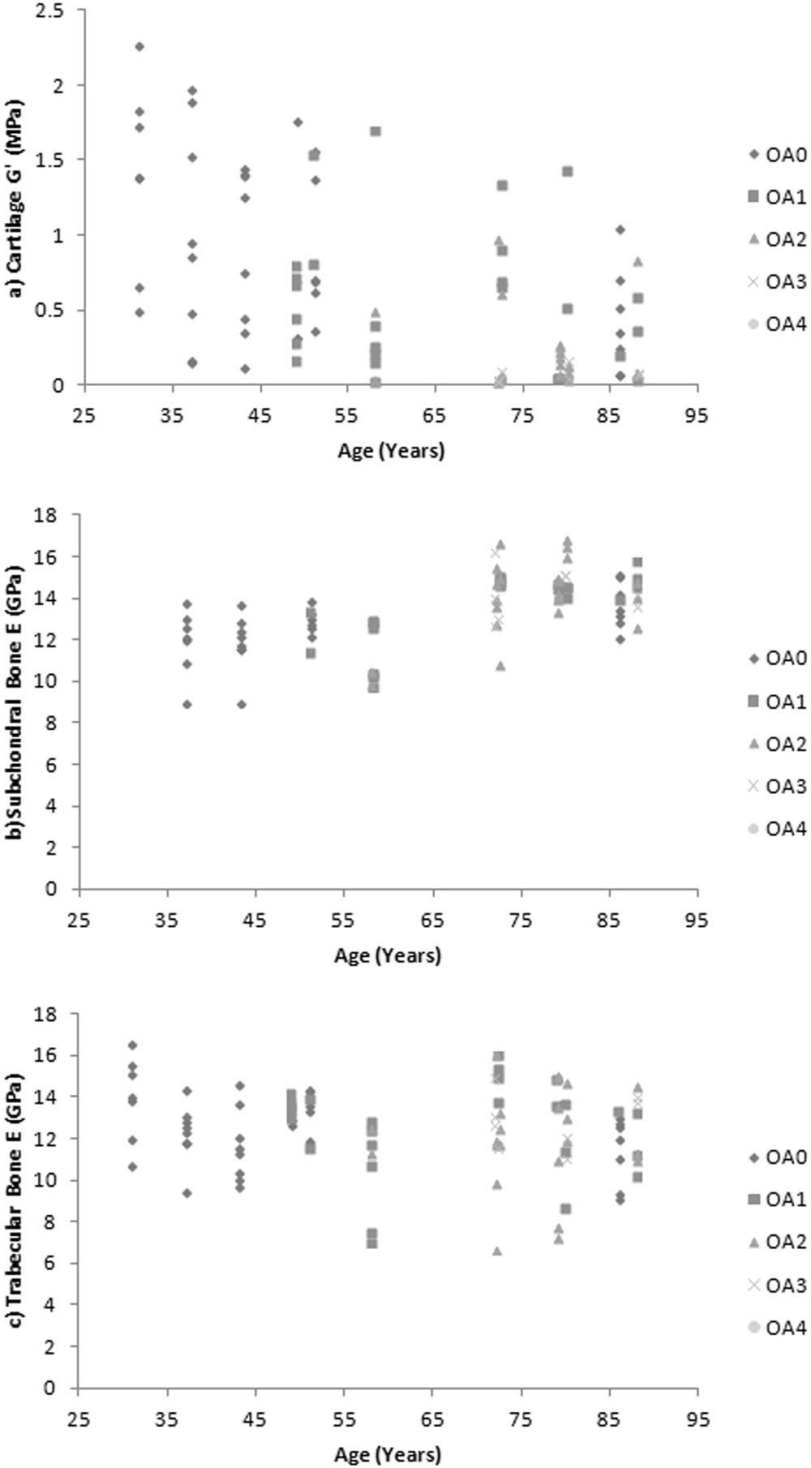
(**a**) Cartilage shear storage modulus (*G*′) (MPa), (**b**) Subchondral bone elastic modulus (*E*) (GPa) and (**c**) Trabecular bone elastic modulus (*E*) (GPa) correlated to age (years) representing n = 8 samples from each cadaver, grouped according to osteoarthritis (OA) International Cartilage Repair Society (ICRS) grade (0–4).

There is also a significant positive correlation between age and overall joint grade of OA (*τ*b = 0.663; *p* = 0.005) (Fig. 3).

### Cartilage and Bone Tissue Interaction

**Figure 4:**
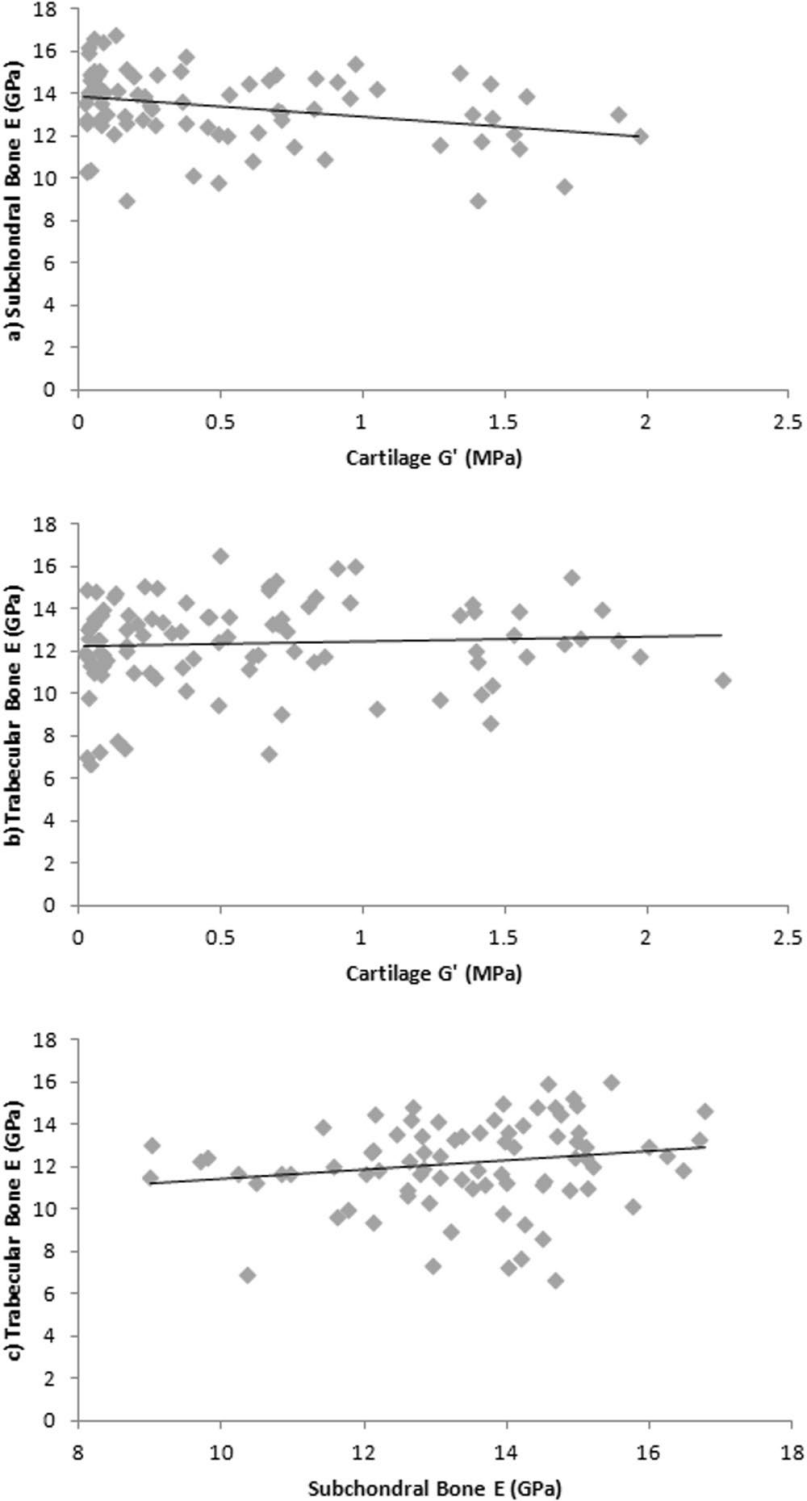
(**a**) Cartilage shear storage modulus (*G*′) (MPa) and subchondral bone elastic modulus (*E*) (GPa) correlation, (**b**) Cartilage shear storage modulus (*G*′) (MPa) and trabecular bone elastic modulus (*E*) correlation (GPa), (**c**) Subchondral bone elastic modulus (*E*) (GPa) and trabecular bone elastic modulus (*E*) (GPa) correlation.

Correlations between the multiple tested tissues can be seen in Figure 4. There is a significant negative correlation between site-matched cartilage *G*′ and subchondral bone *E* (ρ = −0.299; *p* = 0.007). However there is no significant correlation between site-matched cartilage *G*′ and trabecular bone *E* (ρ = 0.105; *p* = 0.309), or site-matched subchondral versus trabecular bone *E* (ρ = 0.210; *p* = 0.061).

### Spatial Distribution of Cartilage and Bone

**Figure 5:**
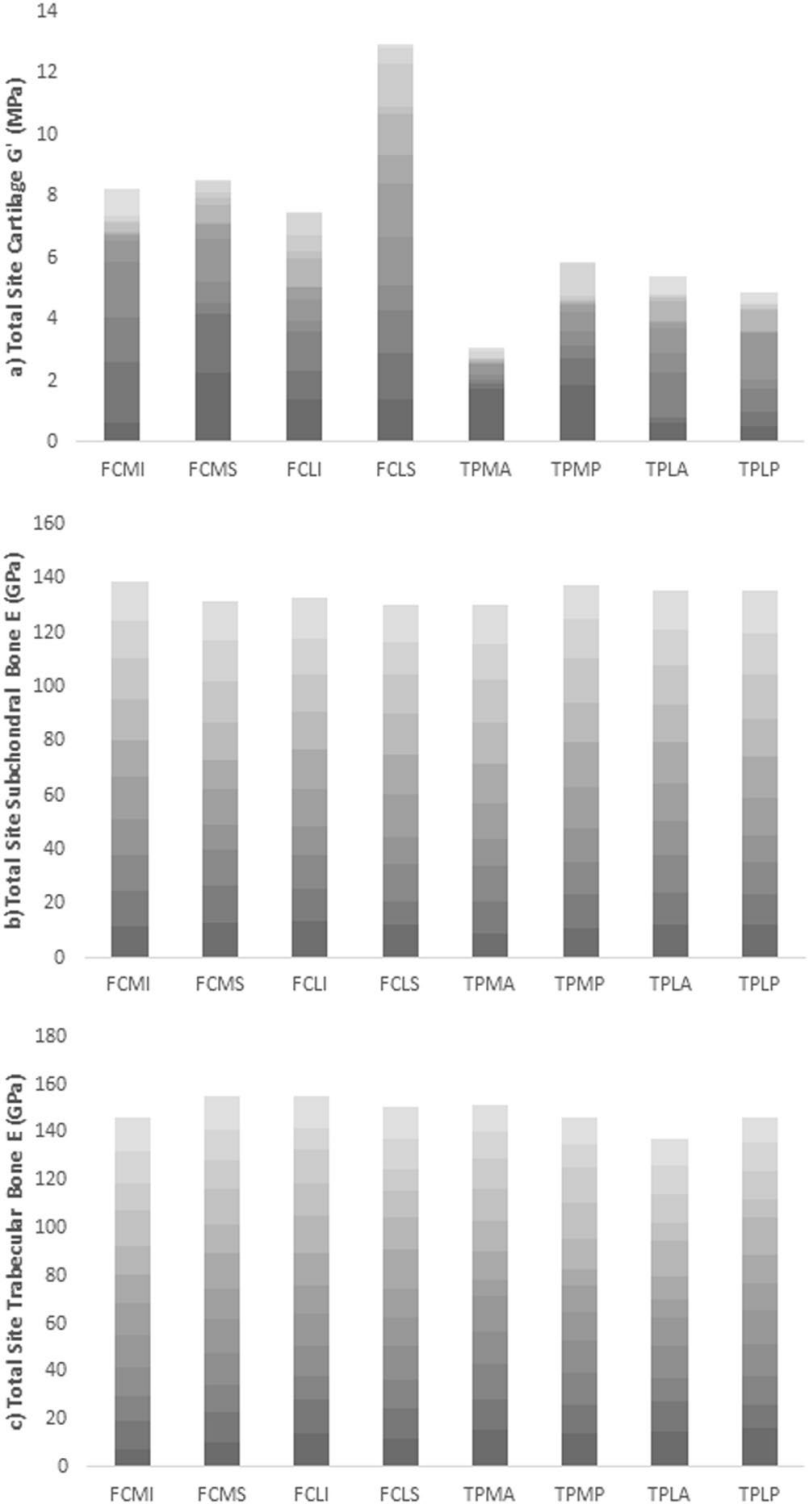
Collated values for (**a**) Cartilage shear storage modulus (*G*′) (MPa), (**b**) Subchondral bone elastic modulus (*E*) (GPa) and (**c**) Trabecular bone elastic modulus (*E*) (GPa) from all cadavers at site specific locations. Femoral condyle medial inferior (FCMI), femoral condyle medial superior (FCMS), femoral condyle lateral inferior (FCLI), femoral condyle lateral superior (FCLS), tibial plateau medial anterior (TPMA), tibial plateau medial posterior (TPMP), tibial plateau lateral anterior (TPLA), tibial plateau lateral posterior (TPLP). Age of cadaver is represented in increasing transparency of colour.

Across the 12 cadavers, combined site mean cartilage *G*′ showed a moderate correlation to spatial locations (*τ*b = −0.500; *p* = 0.083) (Fig. 5). Differences were most common between the mean of femoral and tibial sites, with the lowest *G*′ found at the TPMA and highest at the FCLS. Lower values of *G*′ were more marked at medial sites. Mean and SD femoral and tibial cartilage *G*′ was 0.77 ± 0.62 and 0.40 ± 0.47 MPa respectively, whilst medial versus lateral *G*′ were 0.53 ± 0.63 MPa and 0.64 ± 0.53 respectively.

Subchondral bone and trabecular bone *E* also varied across site-specific locations but no consistent patterns or differences were seen at any one particular site. Mean and SD femoral and tibial subchondral bone *E* was 13.34 ± 1.69 and 13.46 ± 1.78 GPa respectively and medial versus lateral samples were 13.46 ± 1.77 and 13.34 ± 1.70 GPa respectively. Mean and SD femoral and tibial trabecular bone *E* was 12.65 ± 1.79 and 12.10 ± 2.36 GPa respectively and medial versus lateral *E* was 12.48 ± 2.02 and 12.27 ± 2.19 GPa respectively.

### Combined Effect of Variables

To consider individual sample material properties both within and between subjects, while adjusting for both age and OA grade as variables, a compound symmetry mixed linear model was used, showing the random effects on individual sample cartilage *G*′ were significantly different between subjects (*p* = <0.001), but not within subjects (*p* = 0.429). This suggests there was no significant difference of within-subject cartilage *G*′. Using these model assumptions, cartilage *G*′ was significantly correlated to age (*p* = 0.003) but not OA grade (*p* = 0.052), when adjusted for one another and within-subject effects. The random effects of subchondral and trabecular bone *E* were also significantly different between subjects (both *p* = <0.001), but not within subjects (*p* = 0.132 and *p* = 0.547 respectively). Subchondral bone *E* was significantly correlated to age (*p* = 0.010), but not OA grade (*p* = 0.892) when adjusted for one another and within-subject effects. Trabecular bone *E* was not correlated to either age (*p* = 0.432) or OA grade (*p* = 0.809).

## Discussion

This study presents the first systematic quantification of changes in the material properties of multiple human knee tissues by applying a single method to a cohort of cadaveric specimens spanning a wide range in age (31–88 years) and disease state (OA ICRS grade 0–4). These results allow us to determine how properties of all tissues change in the absence of confounding factors of variation of donor demographics and testing methods between studies for the first time (Figs. 1–5). Spatial sampling of multiple tissues also allows us to assess these correlations at the sub-joint level, which is crucial given suggestions of preferential regional development and progression of OA^14^ as well as local changes to tissue morphology and structure thought to be associated with medial compartment mechanical loading of the human knee during habitual locomotion^15^.

A number of previous studies have reported the material properties of healthy human knee joint articular cartilage [e.g.^16,17^] and compared data from healthy samples to those with OA [e.g.^11,12,18–20^]. These studies consistently report a decline in modulus in the presence of disease as an independent variable, which correlates with the statistically significant and highly correlated^21^ negative relationship found here (Fig. 2). Healthy and OA grade 1 human knee joint cartilage *G*′ has been reported between 0.07–6.7 MPa assuming a Poisson’s ratio of 0.46^22^, whilst OA grades 2–3 samples fall between 0.07–0.17 MPa [e.g.^11,12,16–20^]. Most recently Robinson *et al*.^23^, found that cartilage *G*′ at tibial and femoral sites in old (69.7 ± 9.3 years) healthy controls was 6.0 ± 1.6 MPa compared to OA samples (4.6 ± 1.8 MPa). However these earlier studies have not categorised samples according to age, or tested a wide span of age and therefore our ability to understand age-related trends and their influence on OA was limited.

The new data generated herein demonstrates clear changes in the material properties of knee joint tissues with ageing as well as in the presence of disease (Figs. 1–3). The absolute *G*′ values reported for healthy and grade 1 OA samples tend to fall towards the lower end of previously reported results (Fig. 2a) whilst values of OA grades 2–4 tend to fall towards the higher end of previously reported results (Fig. 2a). Variation across previous studies may be due to different testing techniques, donor demographics and preservation and storage methods, which make it challenging to accurately compare data. Importantly, some previous studies and the data generated herein focus primarily on the intrinsic viscoelastic response of cartilage which has been shown to functionally identify surface changes in the presence of early OA^24^. Whilst there is a body of literature also exploring the poroelastic response of cartilage considering the fluid-flow mechanics [e.g.^25,26^], such measurements are outside the scope of the current research. Interestingly, when determining the changes in cartilage *G*′ in a multi-variable analysis, this was correlated to age but not OA grade (*p* = 0.052) when adjusted for one another and the dependence effect of multiple samples per donor. This suggests that ageing has a more prominent effect on cartilage *G*′ than OA grade.

Our study also found evidence for a linear increase in subchondral bone *E* with increasing age (Fig. 1) and OA (Fig. 2). Therefore this data demonstrates, for the first time, a significantly correlated relationship between a change in site-matched cartilage and subchondral bone material properties (Fig. 4). These findings provide direct support for conceptual representations of cartilage and subchondral bone as a single functional unit^6^. Values between 22.0–25.8 GPa have previously been reported for healthy cortical bone *E* from the human knee joint^27^, which are relatively higher than the average samples means across the whole joint with values reported in this study of 11.12–15.33 GPa. However more recently Zuo *et al*.^28^, characterised tissue level mechanical strength of the subchondral bone in OA samples and found higher stiffness values in lamellae of grade 4 samples (17.33 ± 3.13 GPa) when compared to grade 1 samples (13.90 ± 2.75 GPa); however there were no healthy controls included in this study. Thus prior to this research (Figs 2b, 3b) it has not been possible to systematically assess OA material property trends in subchondral bone. Specifically, in the current study older cadavers with OA had higher subchondral bone *E* when compared to healthy aged-matched controls (Fig. 3), further supporting the involvement of subchondral bone in the presence of disease. Endochondral ossification is observed with advancing OA and may cause mechanical stiffening of the subchondral bone^29^, which could account for the increase in *E* with increasing grade of OA (Fig. 2). Our multi-variable analysis also correlated a change in subchondral bone *E* to age, but not OA grade when adjusting for one another, indicating, as with cartilage *G*′, that age has a more prominent effect on subchondral bone *E* than increasing OA grade, but it is difficult to isolate these variables as they usually happen concurrently.

Previous research has also suggested that a change in the density and separation of trabecular bone occurs in the presence of OA^8,9^; however due to inconsistencies in cadaver demographics and variation in testing methods it was previously impossible to gauge how trabecular *E* changes with age or disease. Human knee joint trabecular bone *E* has previously been reported with values between 0.002–1.15 GPa [e.g.^30-33^] spanning three orders of magnitude. It should be noted that these studies represent varying testing methods and tip geometries which can account for some variation in results; however this concurrently makes inter-study comparison between cohorts challenging. Data generated herein shows no systematic change in material properties (ICRS 0: 12.33 ± 3.04 GPa; ICRS 4: 12.07 ± 1.83 GPa) (Figs 1,2), suggesting that changes seen in the presence of OA^8,9^ may be limited to structural adaptations. Further supporting this, our multi-variable analysis showed no correlation of trabecular bone *E* to age or OA when adjusted for one another.

An additional notable finding here which may contribute to varying results from within and between subject analysis, is the relative high level of variability in material properties in all three tissue types, and in particular cartilage, within cadavers of all genders, ages and disease status (Figs 1,2). No obvious or systematic trends in the magnitude of variability with increasing age or OA were identified in the data. The heterogeneous nature of the extracellular matrix of articular cartilage is influenced by variations in composition, structure and vascularity at the micro-level where cartilage material property variability within one specimen at different localities has previously been identified^34^. This strengthens the need to represent such structures locally with interchangeable material properties.

Furthermore the geometry, density and spatial locality plays a role in the variability of bone material properties^35^. The functional importance of spatial heterogeneity in material properties has been conceptually demonstrated in computer simulations of joint mechanics. For example, Mononen *et al*.^36^, represented cartilage as a heterogeneous tissue, varying *E* accordingly to healthy and OA spatial material properties. Regions with OA, and therefore a reduced cartilage *E*, had increased tissue deformation and strain and significantly altered contact and pore pressures, where stresses increased at the interface between healthy and OA tissue^36^. Herein site specific cartilage material property differences exist in individual cadavers (Fig. 5) with absolute differences of up to 1.77 MPa equivalent to a relative difference of 461.2%. Therefore with the current data in mind this suggests a more local approach should be considered in attempts to understand the mechanical function of knee joint tissues, particularly in the presence of OA (Fig. 2).

The data presented in this study demonstrates that OA affects medially located samples more than laterally located ones. The individual ICRS grading of cartilage samples along with *G*′ also suggests preferential development of OA medially, which is consistent with current diagnostic literature^14^. Additionally, motion analysis of healthy individuals also shows increased loading during gait on the medial femoral-tibial joint compared to lateral^15^ as well as increased cartilage strains^37^. This is highly suggestive of a causative link between habitual joint loading and the suggested increase in medial OA seen within the current study. Medial femoral condyle cartilage *G*′ declines with ageing; however such differences are not seen between medial and lateral samples in young healthy cadavers (Fig. 5a & Supplementary Material 1). Interestingly, regional development of OA has previously been applied in finite element (FE) models showing medial femoral condyle OA may create potential failure regions in the lateral condyle^36^. With the current data in consideration this would suggest that a decline in material properties seen in this study in ageing and with the presence of OA may be related to regional joint loading. Of note, cadaver BMI, which may affect magnitude of joint loading, was analysed in the current study against cartilage *G*′, subchondral bone *E* and trabecular bone *E*, although no correlations were found, likely due to low sample numbers.

Spatially correlated material properties (Fig. 5) are practically important for the assessment of OA and resultant interventions. Developing targeted OA therapies relies on understanding alterations of multiple tissues involved in whole-joint function^38^. As suggested by Wen *et al*.^39^, alterations in OA therapies will come from a more in-depth knowledge of the role subchondral bone plays in disease progression, which may include physical therapy, pharmaceuticals, or the development of biomimetic materials. Bisphosphonates such as alendronate inhibit bone remodeling and as a consequence reduce cartilage degeneration in animal experimental models^40^. With the current study supporting the role of an increase in bone to a decrease in cartilage mechanical stiffness (Fig. 4), such therapeutic interventions may be introduced in the presence of OA in an attempt to inhibit disease progression. Applications that rely on material property data such as polymer hydrogels are also increasingly being used to mimic viscoelastic properties of articular cartilage due to their structural similarities^41,42^. Tissue engineering including repair, replacement and regeneration of cellular scaffolding using these biomimetic materials should be based on accurate material properties sourced from healthy spatially distributed cartilage.

Our study has, for the first time, provided novel material property data across a wide span of age and OA grade for site matched cartilage and bone from varying localities in the human knee joint. This data demonstrates that cartilage and bone material properties alter in a synergistic relationship during ageing and disease, where a decrease in cartilage *G*′ is accompanied by an increase in subchondral bone *E*. However this relationship appears to be isolated to the subchondral bone and not the trabecular structure despite morphological changes known to occur during disease^8,9^. Furthermore cartilage and subchondral bone material properties are also strongly correlated to age and OA grade independantly, whilst changes in cartilage are also site dependent. Medial preferential development of OA was also noted where cartilage *G* was strongly correlated to site dependency. This may suggest higher mechanical loading previously observed is a causative link to disease progression. This clinically relevant data can now be applied therapeutically via physical therapy, pharmaceuticals or the development of biomimetic materials where a subject- or cohort-specific approach would be more biologically representative.

## Methods

### Specimens

Fresh-frozen human knee joints (n = 12) were sourced aged 31–88 years (4 female, 8 male). Specific cadaver demographics can be seen in Table S1 (Supplementary Material 2), including height, weight, body mass index (BMI) and cause of death. All cadaveric specimens underwent one freeze-thaw cycle prior to dissection, which has been shown to cause no significant change to integrity of tissues [e.g.^43,44^].

**Figure 6:**
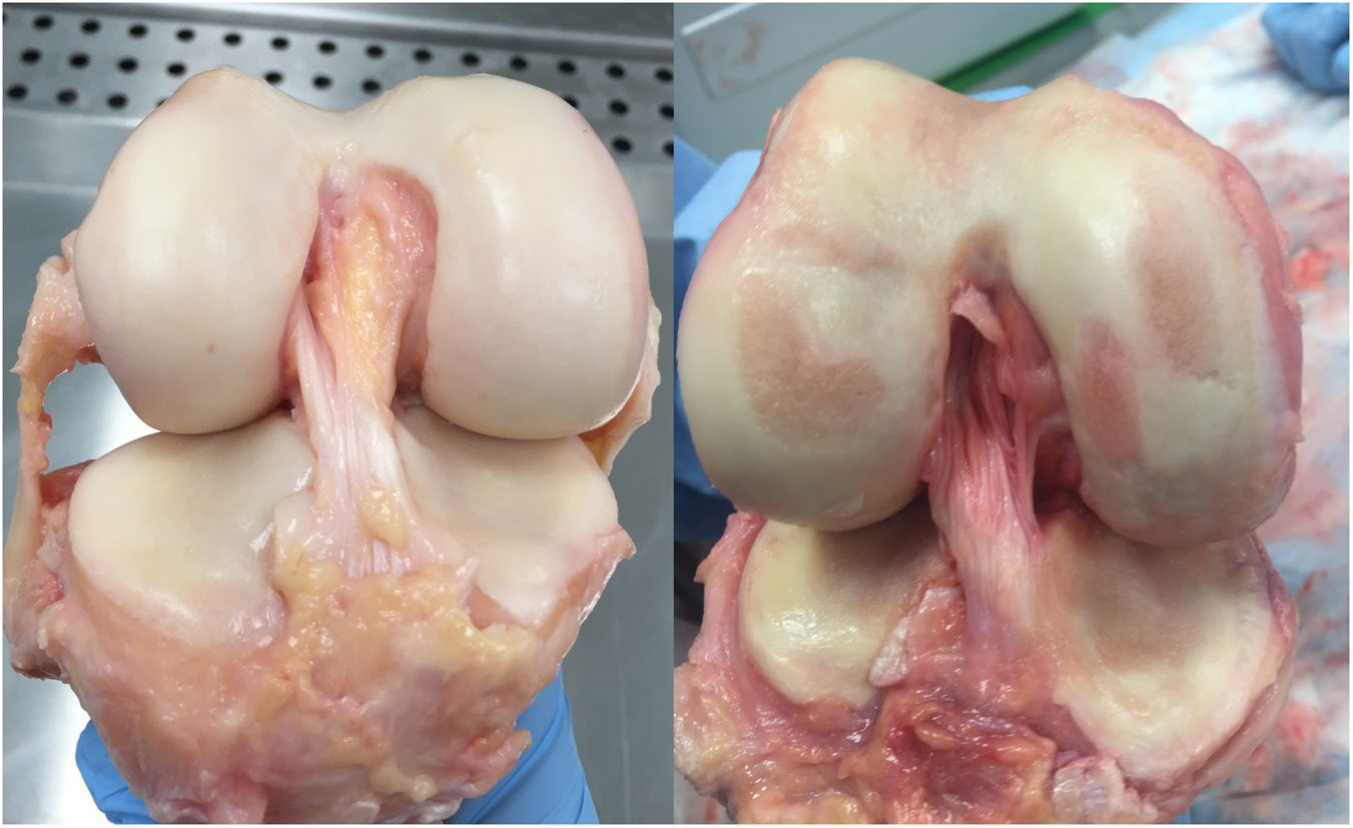
Photographs of young (43 years) healthy (left) and old (88 years) osteoarthritic (right) knee joint specimens.

Individual samples dissected from each cadaver (n = 8 samples per tissue type from each cadaver) were graded for OA using the International Repair Cartilage Society (ICRS) grading system, which is defined in Table S2 (Supplementary Material 2). The cadaveric knee joints were photographed and blind graded by two individuals at a later date three times, one week apart, with the mean score used. Example photographs from one young healthy and one old OA cadaver knee joint can be seen in Figure 6. Photographs from each cadaver can be seen in Figures S1–S12 (Supplementary Material 2).

Eight articular cartilage, eight subchondral bone and eight trabecular bone samples from each of the 12 cadavers were extracted using a low speed oscillating saw (deSoutter Medical, Bucks, UK). Samples were extracted from the following localities: femoral condyle medial inferior (FCMI), femoral condyle medial superior (FCMS), femoral condyle lateral inferior (FCLI), femoral condyle lateral superior (FCLS), tibial plateau medial anterior (TPMA), tibial plateau medial posterior (TPMP), tibial plateau lateral anterior (TPLA) and tibial plateau lateral posterior (TPLP).

### Cartilage

The overlying cartilage (n = 96 samples (n = 8 per cadaver)) was separated from the subchondral bone using a scalpel blade. Cartilage samples were fully submerged in phosphate buffered saline (PBS), transferred on ice and stored at 3–5 °C until testing. All cartilage samples were tested within 72 hours post dissection to avoid any change to material properties^45^.

### Dynamic Nanoindentation Testing

Dynamic nanoindentation (G200 Nanoindenter, Keysight Technologies, Chandler, AZ, USA) was used to obtain the complex shear modulus (G*) of articular cartilage at the micro level. The indenter was equipped with an ultra-low load DCM-II actuator utilising a Continuous Stiffness Measurement (CSM) module and a flat-ended cylindrical 100 µm punch tip (Synton-MDP Ltd, Nidau, Switzerland). Samples were partially submerged in PBS during testing through mounting in a custom-made liquid cell holder measuring a 1 cm radius and 2 mm deep well. Spatially correlated indentation locations (>100 µm spacing between each indentation) were randomly chosen under the optical microscope to achieve 10 measurements per individual sample.

The calculation of shear storage modulus (*G*′), shear loss modulus (*G*′′) and the loss factor (tan delta (δ)) (i.e. ratio of *G*′′/*G*′) were applied following each indentation by assuming a Poisson’s ratio of 0.46^22^. The theoretical basis is detailed elsewhere^46-49^ and has been applied using this method previously^43^, and is briefly outlined here.

Complex shear modulus (G*) is calculated by adding *G*′ (real intrinsic elastic component) to *G*′′ (imaginary viscous component):

1 $${G}^{\ast }=G^{\prime} +iG^{\prime\prime} $$

Sneddon’s analysis is used to calculate the shear storage modulus using the Poisson’s ratio (*v*), contact stiffness (*S*) and tip diameter (*D*), based on using a flat cylindrical punch:

2 $$G^{\prime} =\frac{S(1-v)}{(2D)}$$

The above components along with contact damping (C*w*) can be used to calculate the shear loss modulus:

3 $$G^{\prime\prime} =\frac{{\rm{C}}w\,(1-v)}{(2D)}$$

Contact stiffness (*S*) is calculated by subtracting the instrument stiffness (*Ki*) from the total measured stiffness (*Ks*):

4 $$S=Ks-Ki$$

Contact damping (C*w*) is calculated by subtracting the instrument damping (C*iw*) from the total measured damping (C*sw*):

5 $${\rm{C}}w={\rm{C}}sw-{\rm{C}}iw$$

The elastic modulus (*E*) was then calculated using the shear storage modulus (*G*′) and Poisson’s Ratio (*v*):

6 $$E=2G^{\prime} (1+v)$$

After the indenter head detected the surface of the sample, a pre-compression of 8 μm was applied until the indenter was fully in contact with the sample. The surface detection was determined by a phase shift of the displacement measurement. In order to accurately detect the surface, the phase shift was monitored over a number of data points. Once the surface detection requirement was fulfilled over the predefined number of data points, the initial contact was determined from the first data point in the sequence. Once the indenter was fully in contact with the sample surface it vibrated at a fixed frequency of 110 Hz (the resonant frequency of the indenter) with 500 nm oscillation amplitude. Contact stiffness and damping were obtained through electromagnetic oscillation sequences. The initial oscillation measured instrument stiffness and damping and these were subtracted from the total measurement to obtain the contact response. Material properties were then obtained during the second oscillation.

After each indentation an adjacent sample holder mounted with 3 M double-sided Scotch tape was indented, in order to clean the tip and prevent the transfer of biological material to subsequent test sites, as this may affect measurements. Following testing of each sample fused silica was indented to ensure the tip remained free from residue. Accuracy of the technique and measurements has previously been evidenced on other compliant homogenous structures^50^.

### Bone

Bone samples (n = 80 subchondral bone, n = 96 trabecular bone (n = 8 per cadaver)) were temporarily stored in 70% ethanol to preserve their physiological state^51^. Note: Subchondral bone samples were unable to be tested for cadaver 1 and 4 due to difficulties in polishing preparation. Samples were then washed in a piezoelectric ultrasonic bath using distilled water and pure ethanol to remove any debris, before being embedded in a low viscosity epoxy resin at a transverse angle as to expose both subchondral and trabecular surfaces. Samples were then grinded with progressive silicon carbide paper (300, 600, 1200, 2400, 4000 grit) whilst under constant water irrigation to remove any debris, and polished with alumni paste to a surface finish on 1 µm and colloidal silica to 40 nm.

### Quasi-Static Nanoindentation Testing

Bone samples underwent quasi-static nanoindentation (G200 Nanoindenter, Keysight Technologies, Chandler, AZ, USA) to determine the nano-mechanical hardness (*H*) and *E*. Samples were examined under the optical microscope to randomly choose ten spatially correlated indents per sample (>100 µm spacing between each indentation). A Berkovich sharp pyramidal tip was utilised (20 nm radius) and a Poisson’s ratio of 0.3^52^ was assumed for bone. A penetration depth of 2000 nm was used for subchondral bone and 1200 nm for trabecular bone with a peak hold time of 30 seconds to factor in any viscoelastic response of tissues^53^. Due to the porous nature of trabecular bone the surface approach distance was set at 2000 nm to address any topographic variation in sample height. For subchondral bone this was set to 1000 nm. Surface stiffness detection was limited to 125 Nm^−1^ and samples were unloaded to 90% and held before final unloading to establish thermal drift, which was set to an acceptance level of 0.15 Nm/s^54^. The nanoindenter was calibrated using fused silica prior and after testing, which has known material property values^55^.

This protocol thus achieves continuous loading and partial unloading of samples with an indenter of known geometry and material properties, with loading and penetration depth precisely measured. This approach allows the calculation of *H* and *E* using an established theory^55^, which is briefly outlined here.

Hardness (*H*) is calculated by dividing the maximum load (*P*) reached at peak penetration depth, by the contact area (*A*):

7 $$H=\frac{P\,{\rm{\max }}}{A}$$

The initial unloading stiffness is calculated as below where *P* is the load and h is the depth and *dP*/*dh* is the slope of the line in tangent to the initial unloading curve in the load-displacement plot.

8 $$S=\frac{dP}{dh}=\frac{2}{\sqrt{{\rm{\pi }}}}{E}_{r}\sqrt{A}$$

The reduced indentation modulus (*E*_r_) is then calculated as below where v and v_i_ represent the sample and indenter Poisson’s ratio respectively, and *E* and *E*_i_ are the sample and the indenter modulus respectively.

9 $$\frac{1}{{E}_{{\rm{r}}}}=\frac{(1-{v}^{2})}{E}+\frac{(1-{v}_{{\rm{i}}}^{2})}{{E}_{{\rm{i}}}}$$

### Statistical Analysis

An a-priori power analysis was performed using G*Power software^56^. A total of 42 samples per tissue type was required to distinguish either an effect size of 0.8 with α error probability of 0.05 and power of 0.95 when determining the relationship between multiple tissue means; or an effect size of 0.5 with α error probability of 0.05 and power of 0.95 for correlations to age, OA grade, spatial distribution and BMI. Normal distribution of all measured individual sample material properties was analysed using a Kolmogorov-Smirnov test accounting for skewness and kurtosis of results. Where data was not significant and therefore normally distributed, homogeneity of variance was analysed using the Levene’s test. Homoscedastic data was then tested for linearity using a two-tailed Pearson’s correlation. Data violating the assumptions of Pearson’s correlation testing were analysed using a two-tailed Spearman’s Rank (SPSS software, Version 22.0, SPSS, Inc., Chicago, IL). Specifically bivariate correlation coefficients with significance to age, OA, spatial distribution and BMI of samples was determined. Individual sample and combined sample mean and standard deviation (SD), and 95% confidence interval (CI) were analysed for each tissue from each cadaver. The overall joint mean material properties were also correlated to age and overall joint OA grade (n = 12), and to sample site (n = 8 locations) using a Kendall’s Tau-b test. Joint means were used to account for within-subject dependence of samples. The effect of within and between-subject variables were analysed using a mixed linear model, combing the effects of both age and OA.

The results primarily focus on the intrinsic viscoelastic *G*′ of cartilage and *E* of subchondral and trabecular bone, as these are the most commonly reported and therefore comparable results. Shear and elastic properties are also most closely linked to tissue function *in vivo*. However to aid a full interpretation of data collected, additional data is also reported within Supplementary Material 1.

All data generated or analysed during this study are included in this published article (and its Supplementary Information files).

Ethical permission for use of this human cadaveric material was sponsored by the University of Liverpool and granted by the NRES (15/NS/0053) who approved all protocols. All experiments were performed in accordance with relevant guidelines and regulations.

## Electronic supplementary material

Supplementary Material 1

Supplementary Material 2

## References

[1] Zhang Y, Jordan JM (2008). Epidemiology of Osteoarthritis. Rheumatic Disease Clinics of North America..

[2] Kiss RM (2011). Effect of severity of knee osteoarthritis on the variability of gait parameters. Journal of Electromyography and Kinesiology..

[3] Hollman JH, Kovash FM, Kubik JJ, Linbo RA (2007). Age-related differences in spatiotemporal markers of gait stability during dual task walking. Gait & Posture..

[4] Hansen U, Masouros S, Amis AA (2006). (iii) Material properties of biological tissues related to joint surgery. Current Orthopaedics..

[5] Manninen P, Riihimaki H, Heliovaara M, Makela P (1996). Overweight, gender and knee osteoarthritis. International Journal of Obesity and Related Metabolic Disorders: Journal of the International Association for the Study of Obesity..

[6] Mahjoub M, Berenbaum F, Houard X (2012). Why subchondral bone in osteoarthritis? The importance of the cartilage bone interface in osteoarthritis. Osteoporosis International..

[7] Burr DB, Gallant MA (2012). Bone remodelling in osteoarthritis. Nature Reviews Rheumatology..

[8] Kamibayashi L, Wyss U, Cooke T, Zee B (1995). Trabecular microstructure in the medial condyle of the proximal tibia of patients with knee osteoarthritis. Bone..

[9] Bobinac D, Spanjol J, Zoricic S, Maric I (2003). Changes in articular cartilage and subchondral bone histomorphometry in osteoarthritic knee joints in humans. Bone..

[10] Madry H, van Dijk CN, Mueller-Gerbl M (2010). The basic science of the subchondral bone. Knee Surgery, Sports Traumatology, Arthroscopy..

[11] Kleemann R, Krocker D, Cedraro A, Tuischer J, Duda G (2005). Altered cartilage mechanics and histology in knee osteoarthritis: relation to clinical assessment (ICRS Grade). Osteoarthritis and Cartilage..

[12] Wilusz RE, Zauscher S, Guilak F (2013). Micromechanical mapping of early osteoarthritic changes in the pericellular matrix of human articular cartilage. Osteoarthritis and Cartilage..

[13] Kuroki K, Cook C, Cook J (2011). Subchondral bone changes in three different canine models of osteoarthritis. Osteoarthritis and Cartilage..

[14] Pelletier J (2007). Risk factors associated with the loss of cartilage volume on weight-bearing areas in knee osteoarthritis patients assessed by quantitative magnetic resonance imaging: a longitudinal study. Arthritis Research & Therapy..

[15] Kumar D, Manal KT, Rudolph KS (2013). Knee joint loading during gait in healthy controls and individuals with knee osteoarthritis. Osteoarthritis and Cartilage..

[16] Shepherd DE, Seedhom BB (1999). The ‘instantaneous’ compressive modulus of human articular cartilage in joints of the lower limb. Rheumatology (Oxford, England)..

[17] Thambyah A, Nather A, Goh J (2006). Mechanical properties of articular cartilage covered by the meniscus. Osteoarthritis and Cartilage..

[18] Hori RY, Mockros L (1976). Indentation tests of human articular cartilage. Journal of Biomechanics..

[19] Franz T (2001). *In situ* compressive stiffness, biochemical composition, and structural integrity of articular cartilage of the human knee joint. Osteoarthritis and Cartilage..

[20] Wang M, Peng Z, Price J, Ketheesan N (2013). Study of the nano-mechanical properties of human knee cartilage in different wear conditions. Wear..

[21] Cohen, J. Statistical power analysis for the behavioural sciences. (Lawrence Earlbaum Associates, 1988).

[22] Jin H, Lewis JL (2004). Determination of Poisson’s ratio of articular cartilage by indentation using different-sized indenters. Journal of Biomechanical Engineering..

[23] Robinson DL (2016). Mechanical properties of normal and osteoarthritic human articular cartilage. Journal of the Mechanical Behavior of Biomedical Materials..

[24] Desrochers J, Amrein M, Matyas J (2012). Viscoelasticity of the articular cartilage surface in early osteoarthritis. Osteoarthritis and Cartilage..

[25] Taffetani M, Gottardi R, Gastaldi D, Raiteri R, Vena P (2014). Poroelastic response of articular cartilage by nanoindentation creep tests at different characteristic lengths. Medical Engineering & Physics..

[26] Nia HT, Han L, Li Y, Ortiz C, Grodzinsky A (2011). Poroelasticity of cartilage at the nanoscale. Biophysical Journal..

[27] Rho J, Tsui TY, Pharr GM (1997). Elastic properties of human cortical and trabecular lamellar bone measured by nanoindentation. Biomaterials..

[28] Zuo Q (2016). Characterization of nano-structural and nano-mechanical properties of osteoarthritic subchondral bone. BMC Musculoskeletal Disorders..

[29] Cox L, van Donkelaar C, van Rietbergen B, Emans P, Ito K (2013). Alterations to the subchondral bone architecture during osteoarthritis: bone adaptation vs endochondral bone formation. Osteoarthritis and Cartilage..

[30] Behrens J, Walker P, Shoji H (1974). Variations in strength and structure of cancellous bone at the knee. Journal of Biomechanics..

[31] Ducheyne P (1977). The mechanical behaviour of intracondylar cancellous bone of the femur at different loading rates. Journal of Biomechanics..

[32] Burgers TA, Mason J, Niebur G, Ploeg HL (2008). Compressive properties of trabecular bone in the distal femur. Journal of Biomechanics..

[33] Zysset P, Sonny M, Hayes W (1994). Morphology-mechanical property relations in trabecular bone of the osteoarthritic proximal tibia. The Journal of Arthroplasty..

[34] Moore A, Burris D (2015). Tribological and material properties for cartilage of and throughout the bovine stifle: support for the altered joint kinematics hypothesis of osteoarthritis. Osteoarthritis and Cartilage..

[35] Zysset PK, Guo XE, Hoffler CE, Moore KE, Goldstein SA (1999). Elastic modulus and hardness of cortical and trabecular bone lamellae measured by nanoindentation in the human femur. Journal of Biomechanics..

[36] Mononen M (2012). Effect of superficial collagen patterns and fibrillation of femoral articular cartilage on knee joint mechanics—A 3D finite element analysis. Journal of Biomechanics..

[37] Adouni M, Shirazi-Adl A, Shirazi R (2012). Computational biodynamics of human knee joint in gait: from muscle forces to cartilage stresses. Journal of Biomechanics..

[38] Goldring SR, Goldring MB (2016). Changes in the osteochondral unit during osteoarthritis: structure, function and cartilage-bone crosstalk. Nature Reviews Rheumatology..

[39] Wen C, Lu WW, Chiu KY (2014). Importance of subchondral bone in the pathogenesis and management of osteoarthritis from bench to bed. Journal of Orthopaedic Translation..

[40] Hayami T (2004). The role of subchondral bone remodeling in osteoarthritis: reduction of cartilage degeneration and prevention of osteophyte formation by alendronate in the rat anterior cruciate ligament transection model. Arthritis & Rheumatism..

[41] Li W, Wang D, Yang W, Song Y (2016). Compressive mechanical properties and microstructure of PVA–HA hydrogels for cartilage repair. RSC Advances..

[42] Wang Q, Hou R, Cheng Y, Fu J (2012). Super-tough double-network hydrogels reinforced by covalently compositing with silica-nanoparticles. Soft Matter..

[43] Peters AE, Comerford EJ, Macaulay S, Bates KT, Akhtar R (2017). “Micromechanical properties of canine femoral articular cartilage following multiple freeze-thaw cycles. Journal of the Mechanical Behavior of Biomedical Materials..

[44] Moon DK, Woo SL, Takakura Y, Gabriel MT, Abramowitch SD (2006). The effects of refreezing on the viscoelastic and tensile properties of ligaments. Journal of Biomechanics..

[45] Changoor A, Fereydoonzad L, Yaroshinsky A, Buschmann MD (2010). Effects of refrigeration and freezing on the electromechanical and biomechanical properties of articular cartilage. Journal of Biomechanical Engineering..

[46] Herbert E, Oliver W, Lumsdaine A, Pharr GM (2009). Measuring the constitutive behavior of viscoelastic solids in the time and frequency domain using flat punch nanoindentation. Journal of Materials Research..

[47] Herbert E, Oliver W, Pharr G (2008). Nanoindentation and the dynamic characterization of viscoelastic solids. Journal of Physics D: Applied Physics..

[48] Sneddon IN (1965). The relation between load and penetration in the axisymmetric Boussinesq problem for a punch of arbitrary profile. International Journal of Engineering Science..

[49] Landau LD, Lifshitz E (1986). Theory of Elasticity, vol. 7. Course of Theoretical Physics..

[50] Moronkeji, K., Todd, S., Dawidowska, I., Barrett, S. & Akhtar, R. The role of subcutaneous tissue stiffness on microneedle performance in a representative *in vitro* model of skin. *Journal of Controlled Release*. (2016). 10.1016/j.jconrel.2016.11.004 27838272

[51] Linde F, Sørensen HCF (1993). The effect of different storage methods on the mechanical properties of trabecular bone. Journal of Biomechanics..

[52] Reilly DT, Burstein AH (1975). The elastic and ultimate properties of compact bone tissue. Journal of Biomechanics..

[53] Chudoba T, Richter F (2001). Investigation of creep behaviour under load during indentation experiments and its influence on hardness and modulus results. Surface and Coatings Technology..

[54] Oyen M (2013). Nanoindentation of biological and biomimetic materials. Experimental Techniques..

[55] Oliver WC, Pharr GM (1992). An improved technique for determining hardness and elastic modulus using load and displacement sensing indentation experiments. Journal of Materials Research..

[56] Faul F, Erdfelder E, Lang A, Buchner A (2007). G* Power 3: A flexible statistical power analysis program for the social, behavioral, and biomedical sciences. Behavior Research Methods..

