# Supplementary material for: The effect of ageing and osteoarthritis on the mechanical properties of cartilage and bone in the human knee joint
Source: Sci Rep. 2018 Apr 12;8:5931. doi: 10.1038/s41598-018-24258-6 (PMC5897376; doi:10.1038/s41598-018-24258-6)
Supplement: Supplementary file 2 — Supplementary Material 2 [file 41598_2018_24258_MOESM2_ESM.pdf]

The effect of ageing and osteoarthritis on the mechanical properties of cartilage and bone in the human knee joint

Abby E. Peters[1,2*], Riaz Akhtar[2], Eithne J. Comerford[1,2,3], Karl T. Bates[1]

[1]Department of Musculoskeletal Biology, Institute of Ageing and Chronic Disease, University of Liverpool, The William Henry Duncan Building, 6 West Derby Street, Liverpool L7 8TX, UK;

[2] Department of Mechanical, Materials and Aerospace Engineering, School of Engineering, University of Liverpool, The Quadrangle, Brownlow Hill, Liverpool, L69 3GH, UK;

[3]Institute of Veterinary Science, Leahurst Campus, University of Liverpool, Chester High Road, Neston, Wirral, CH64 7TE, UK.

*Corresponding author email: abby.peters@liverpool.ac.uk

Table S1. Cadaver demographics.

	Age	Gender	Race	Height (cm)	Weight (kg)	BMI	Cause of Death
Cadaver 1	31	Female	Not known	172.7	47.2	15.81	Cardiac arrest
Cadaver 2	37	Female	White	160.0	79.4	31.00	Intracerebral haemorrhage; Severe hypertension
Cadaver 3	43	Female	White	170.2	64.4	22.24	Metastatic cervical carcinoma
Cadaver 4	49	Male	White	175.3	58.5	19.05	Not known
Cadaver 5	51	Male	White	182.9	104.3	31.19	Cardiac arrhythmia; Coronary artery disease
Cadaver 6	58	Male	White	188.0	84.8	24.01	Gunshot wound of head and neck
Cadaver 7	72	Male	Puerto Rican	162.6	70.3	26.60	Atherosclerotic heart disease of native coronary
Cadaver 8	72	Male	White	167.6	72.6	25.82	Debility; Alzheimer's disease
Cadaver 9	79	Male	White	172.7	72.1	24.17	Acute myocardial infarction; Coronary artery disease
Cadaver 10	80	Male	White	182.9	83.9	25.09	Myocardial infarction; Cardiac arrest; Hypertension
Cadaver 11	86	Female	White	165.1	63.5	23.29	Respiratory failure; Pneumonia
Cadaver 12	88	Male	White	177.8	68.0	21.52	Natural causes; Unspecified

Table S2. International Cartilage Repair Society (ICRS) Grading.

ICRS Grade	Description
0<br>Normal	No lesions fissures or cracks.
1<br>Nearly Normal	Superficial lesions. Soft indentation and/or superficial fissures and cracks.
2<br>Abnormal	Lesions extending down to <50% of cartilage depth.
3<br>Severely Abnormal	Cartilage defects extending down >50% of cartilage depth as well as down to calcified layer and down to but not through the subchondral bone. Blisters are included in this Grade.
4<br>Severely Abnormal	Cartilage defects extending down >75% of cartilage depth as well as down to calcified layer and through the subchondral bone. Blisters are included in this Grade.

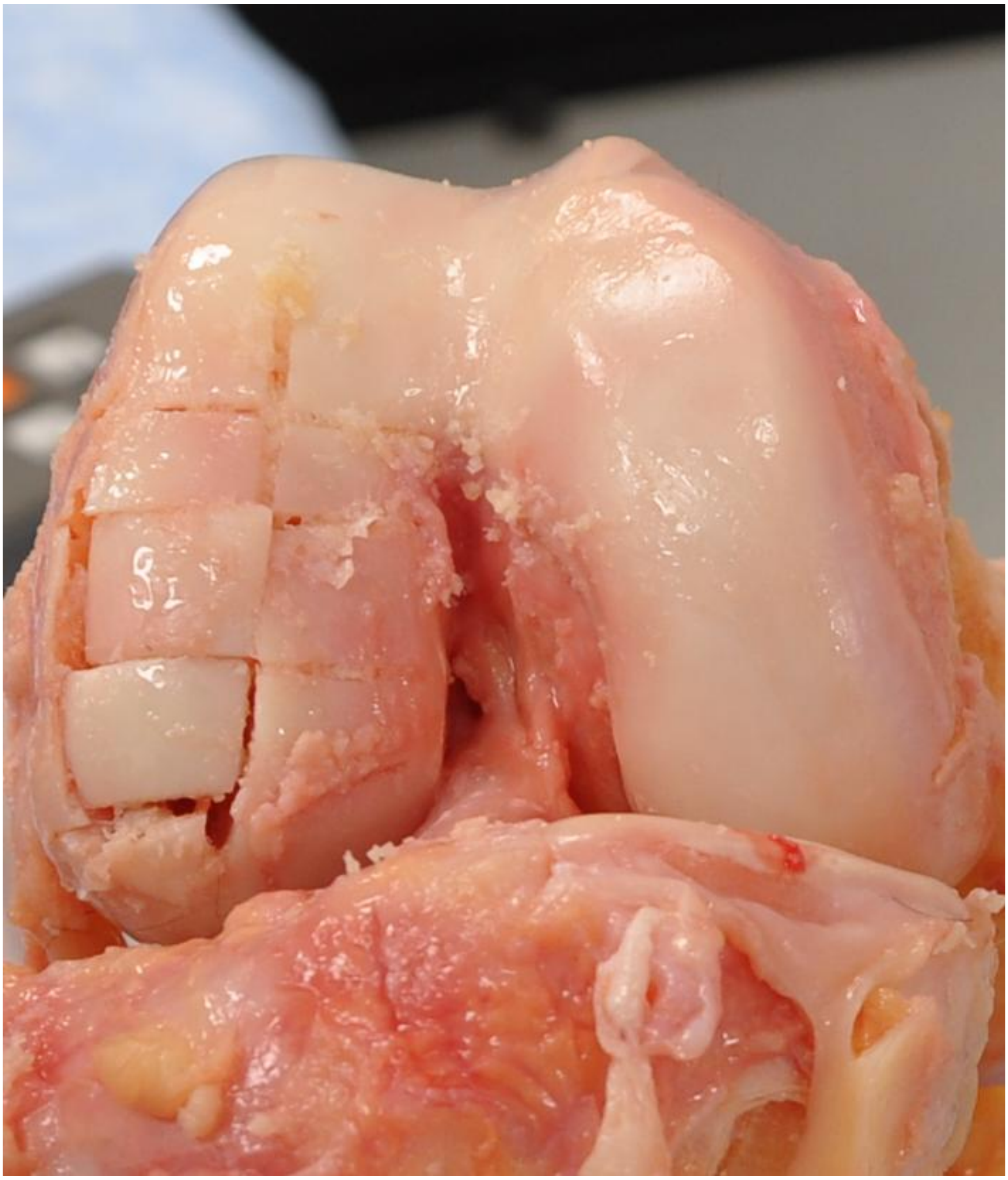

Figure S1. Cadaver 1, 31 years.

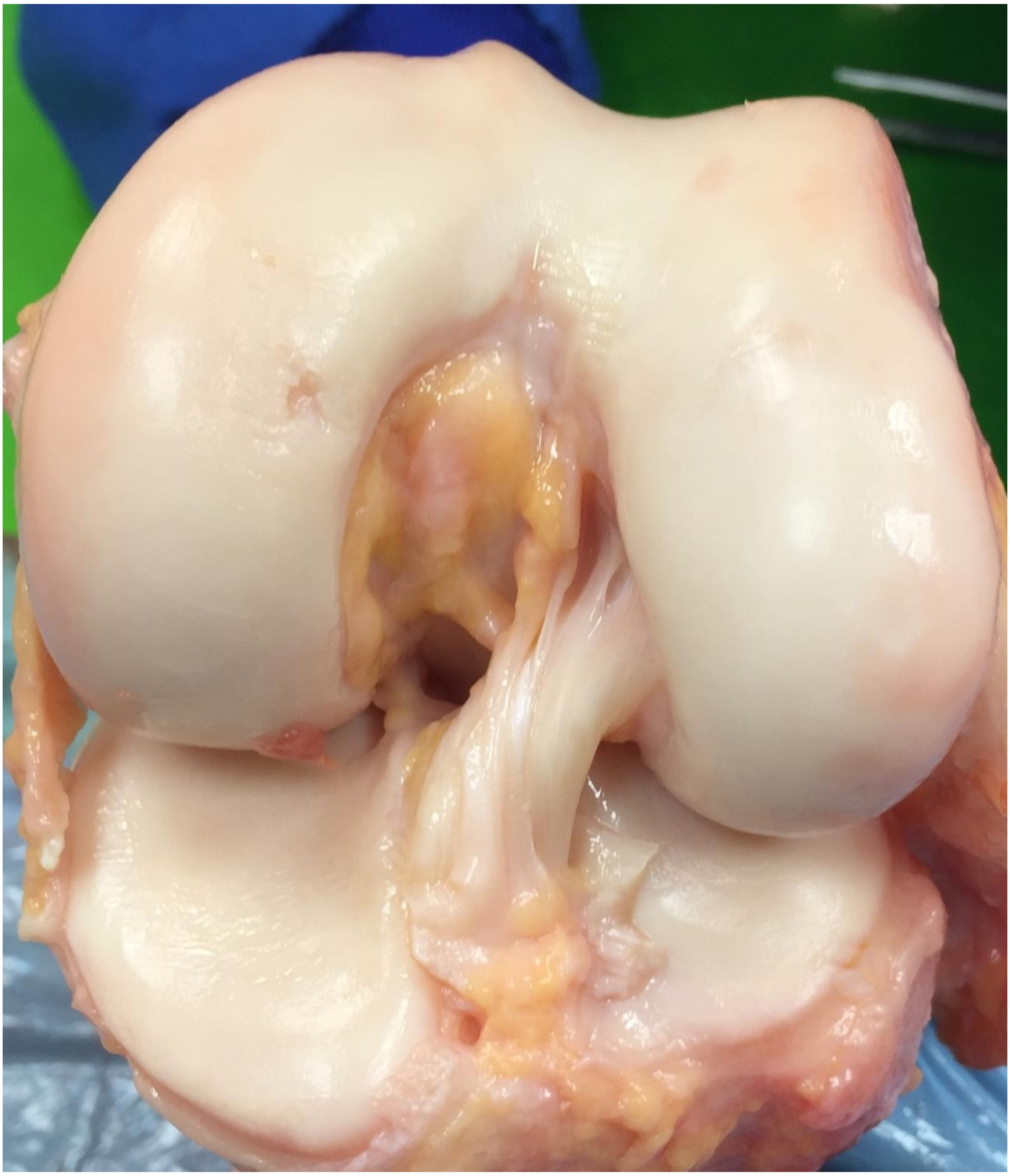

Figure S2. Cadaver 2, 37 years.

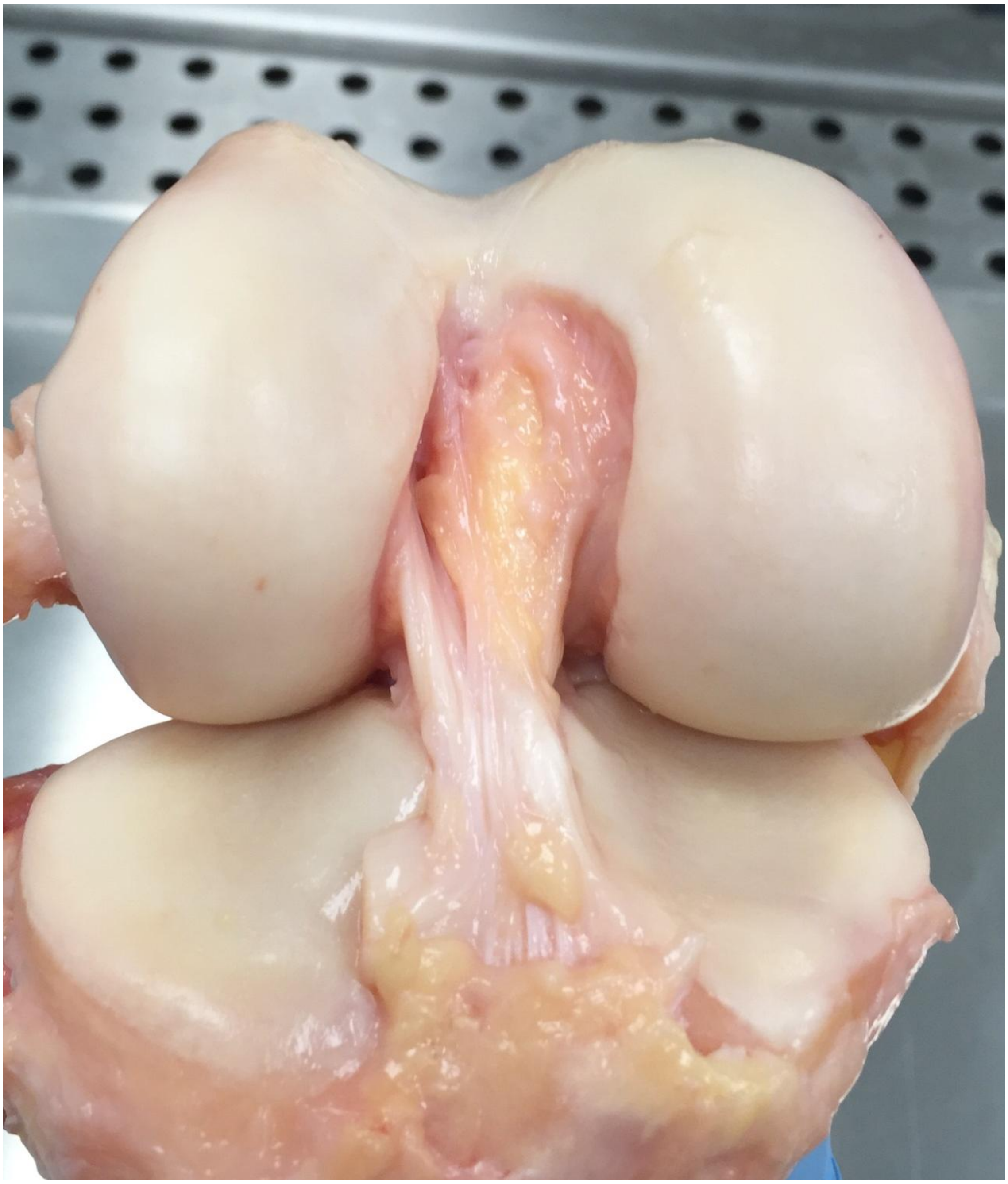

Figure S3. Cadaver 3, 43 years.

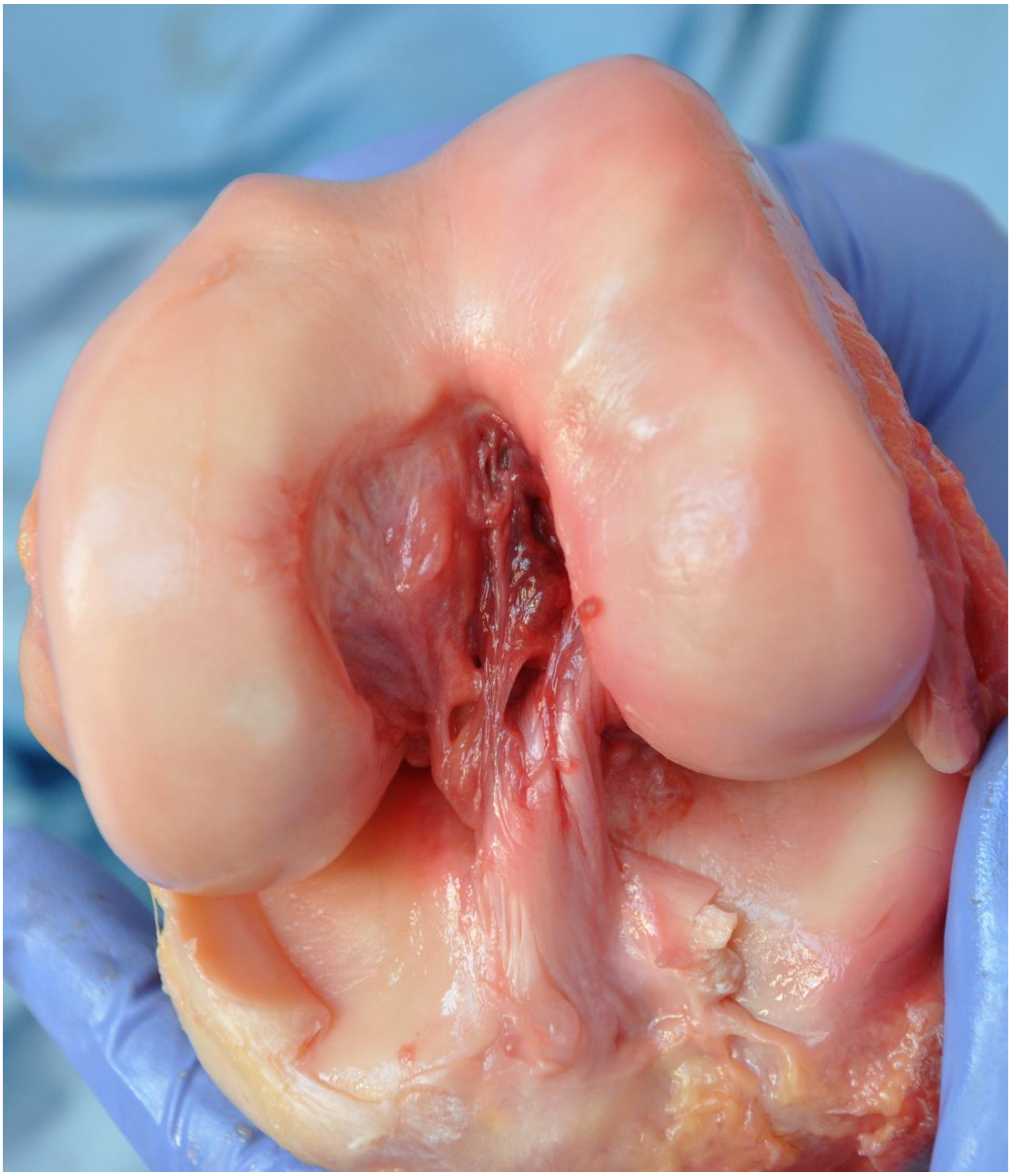

Figure S4. Cadaver 4, 49 years.

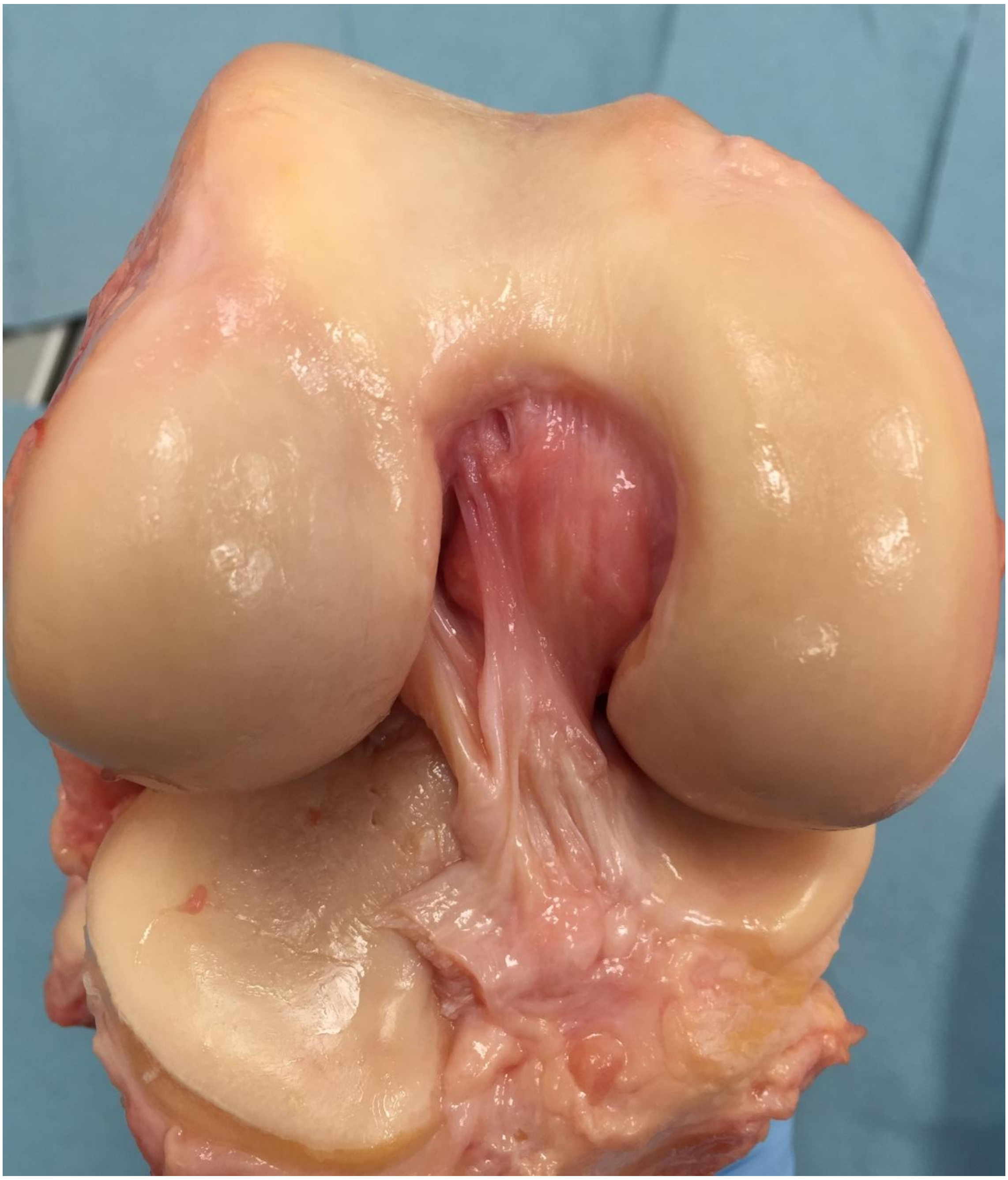

Figure S5. Cadaver 5, 51 years.

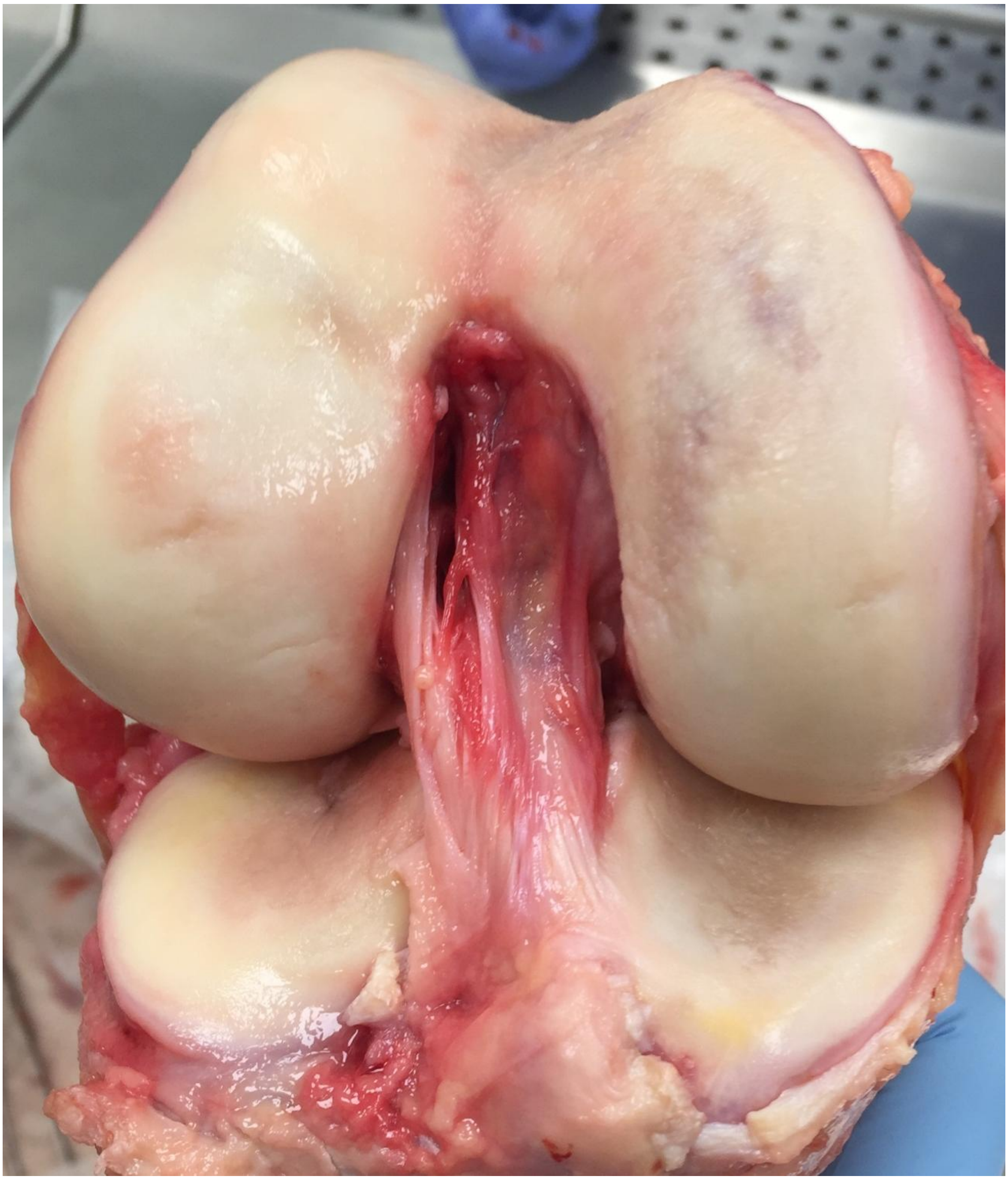

Figure S6. Cadaver 6, 58 years.

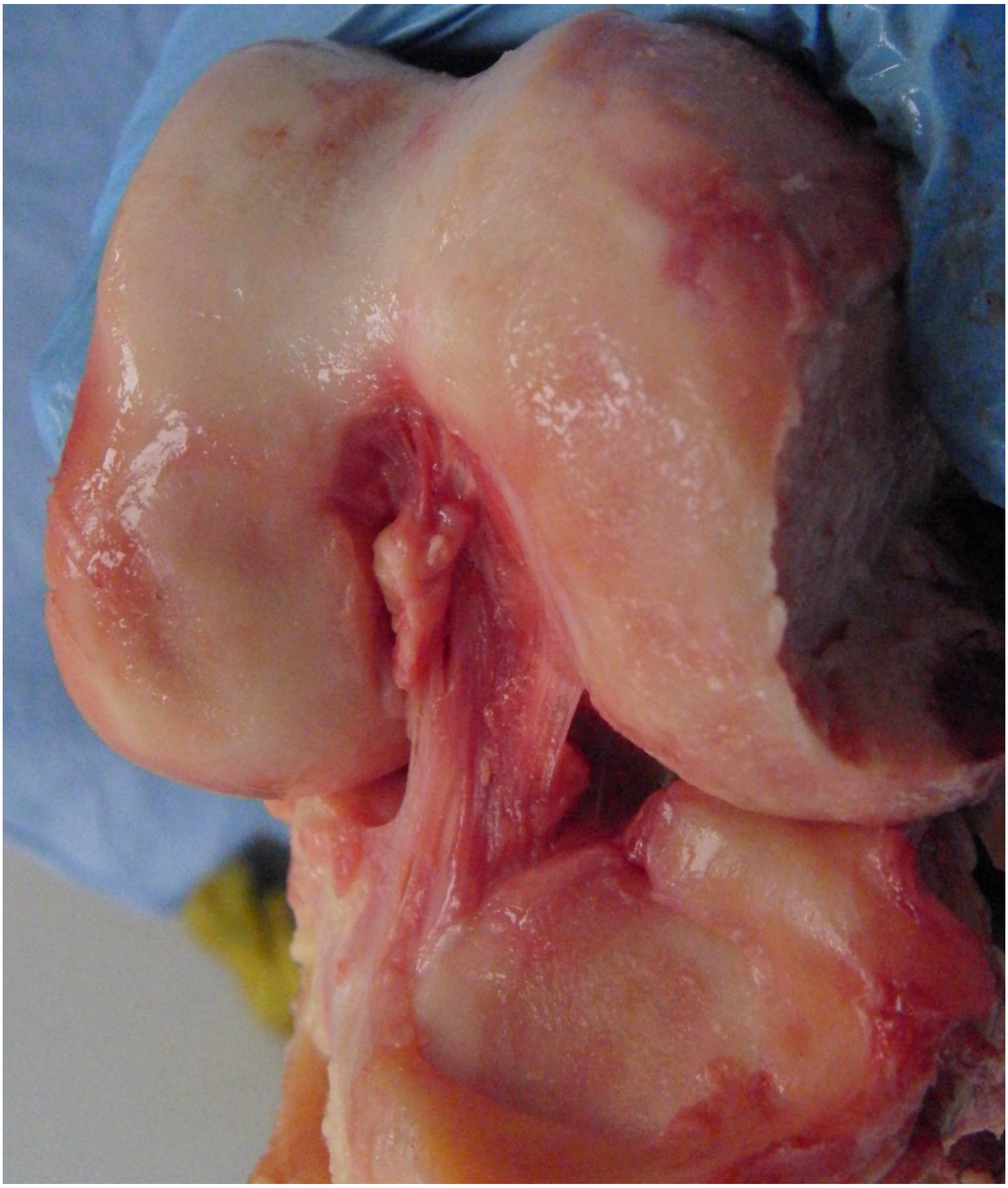

Figure S7. Cadaver 7, 72 years.

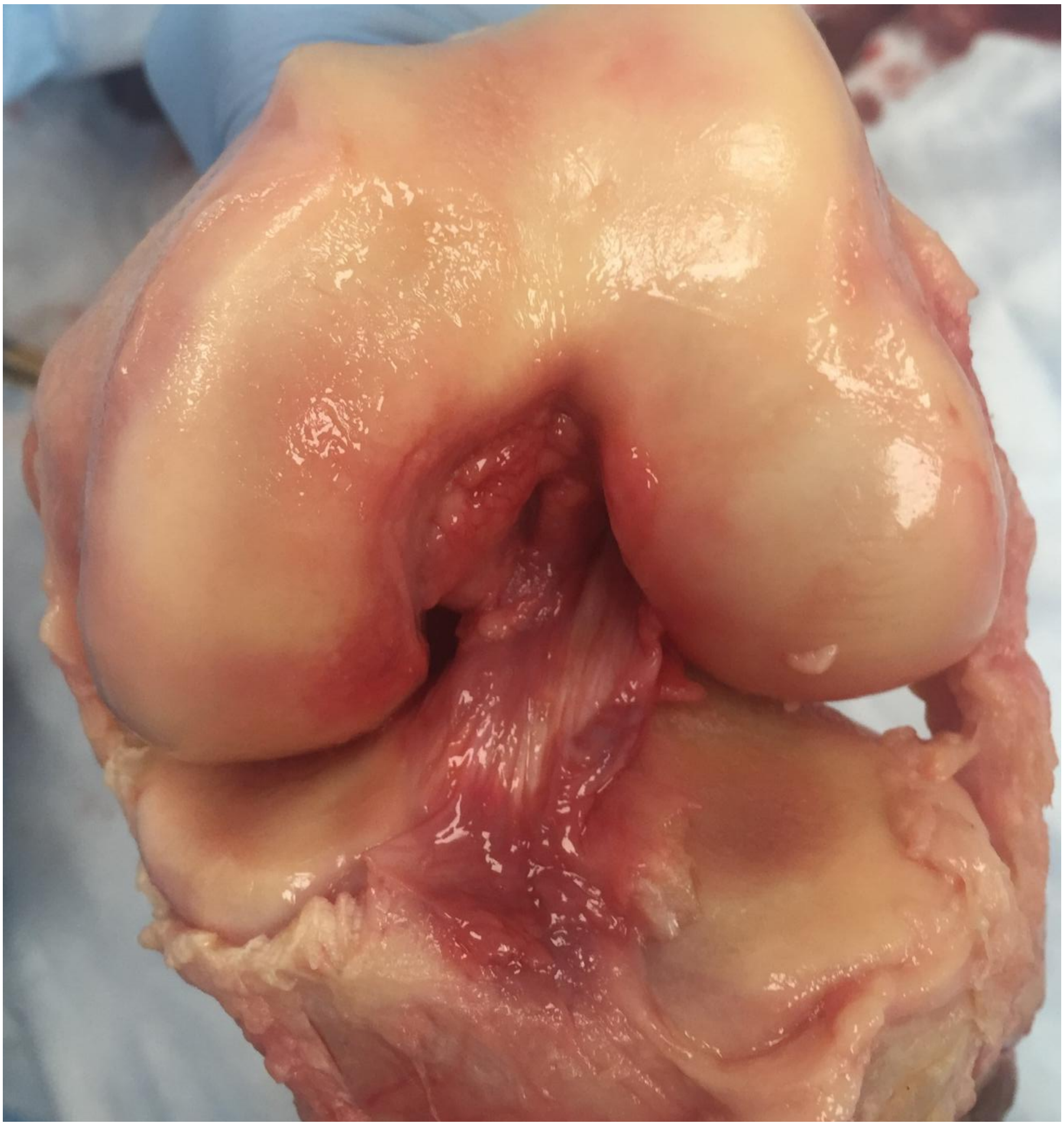

Figure S8. Cadaver 8, 72 years.

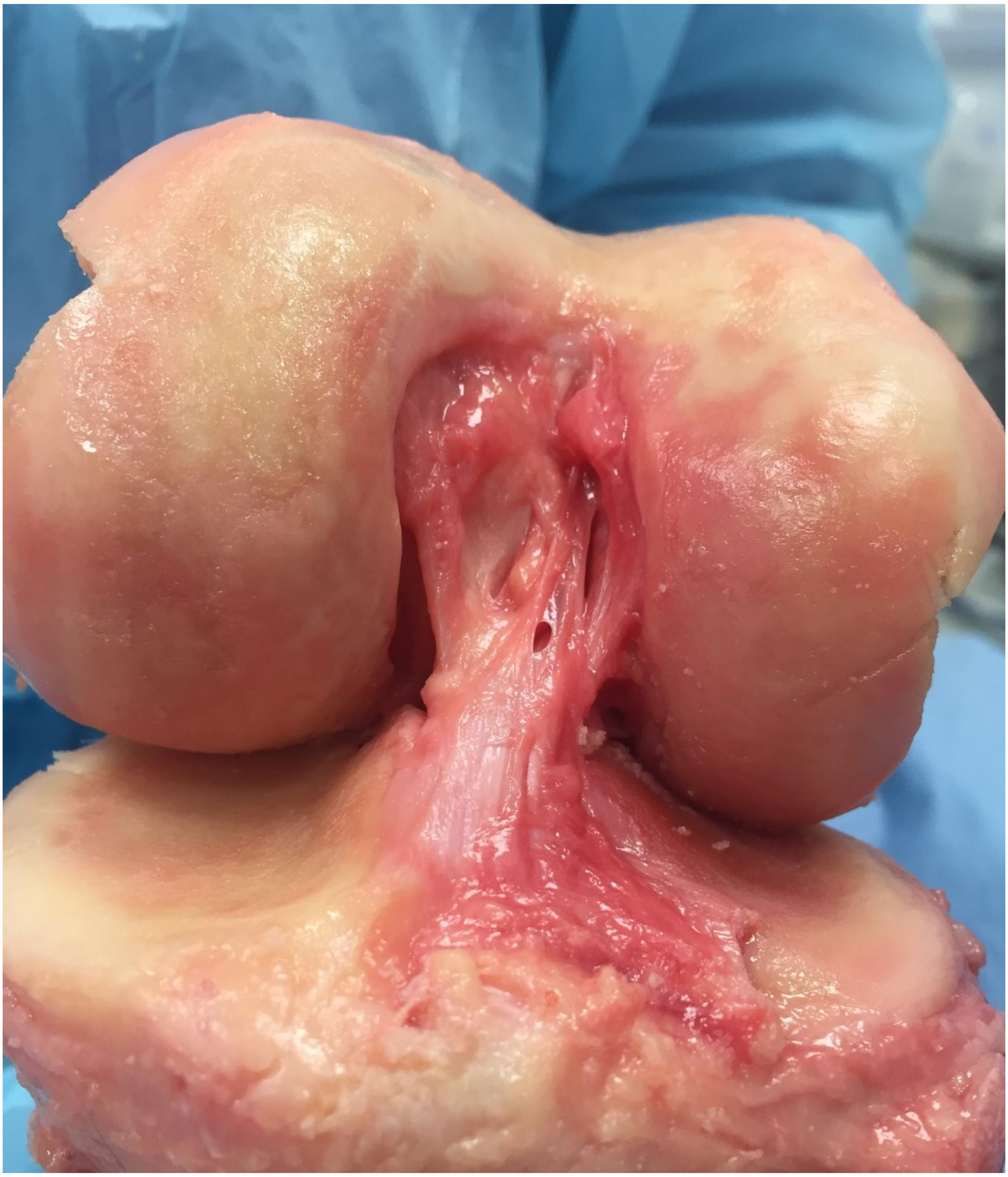

Figure S9. Cadaver 9, 79 years.

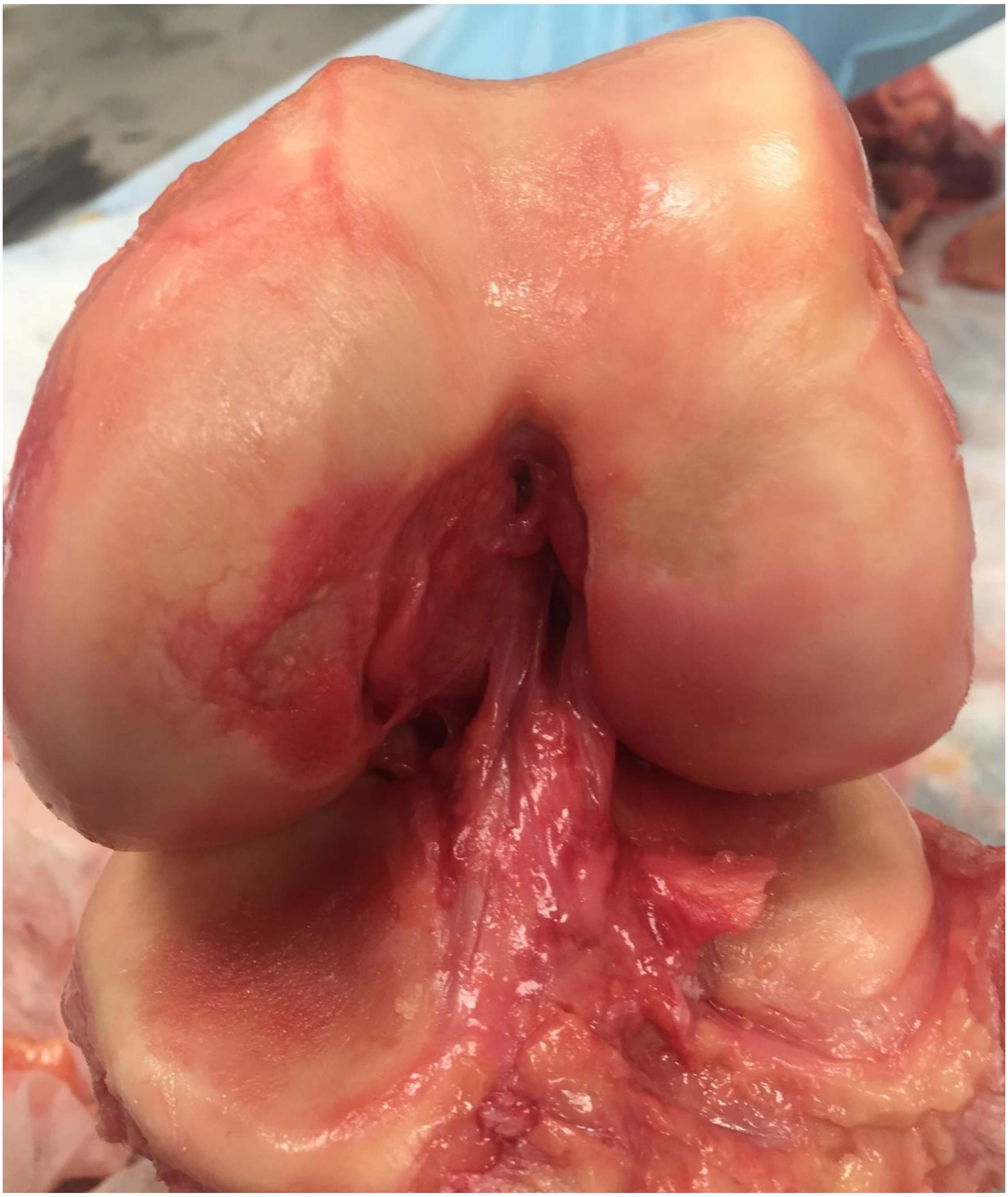

Figure S10. Cadaver 10, 80 years.

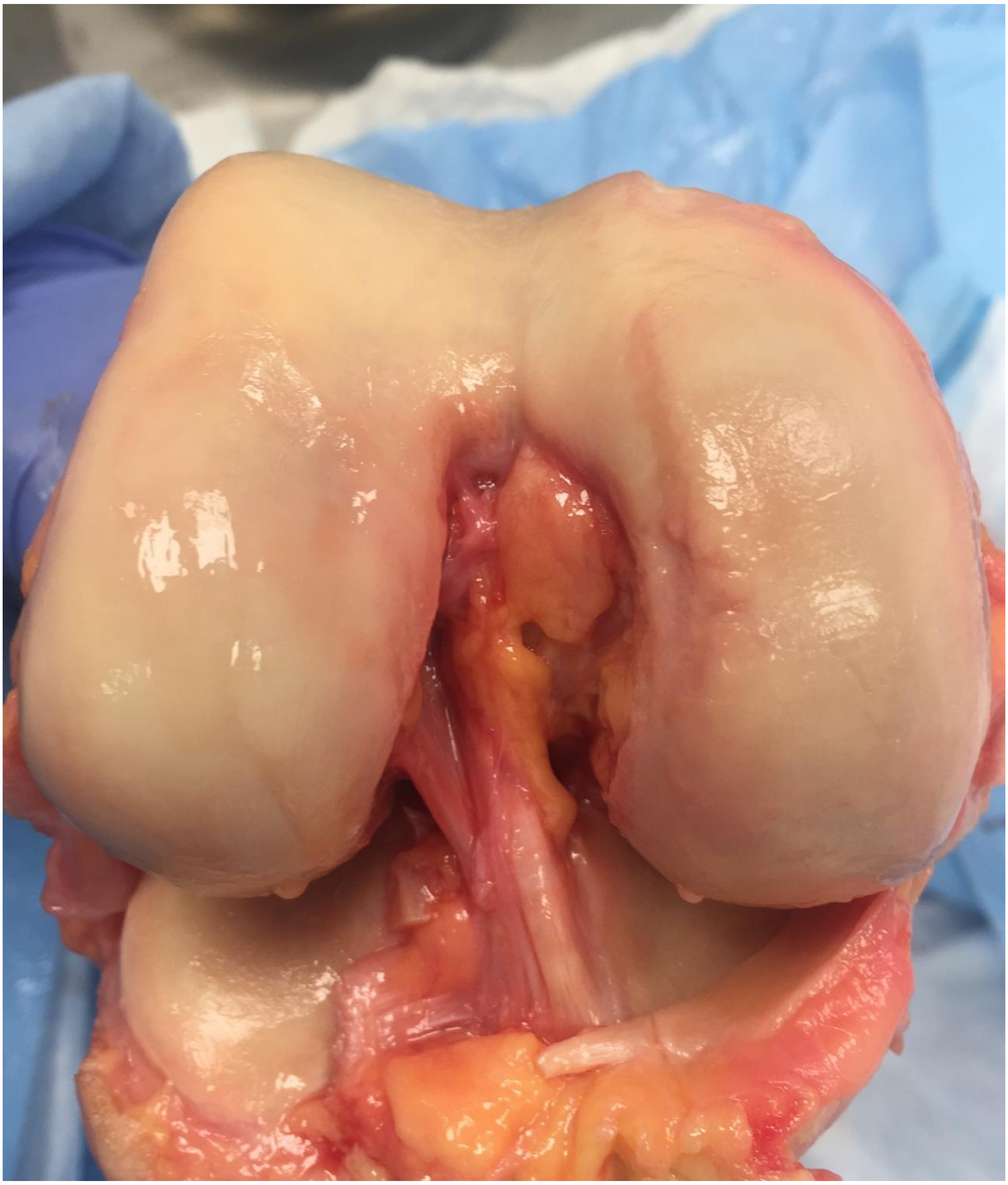

Figure S11. Cadaver 11, 86 years.

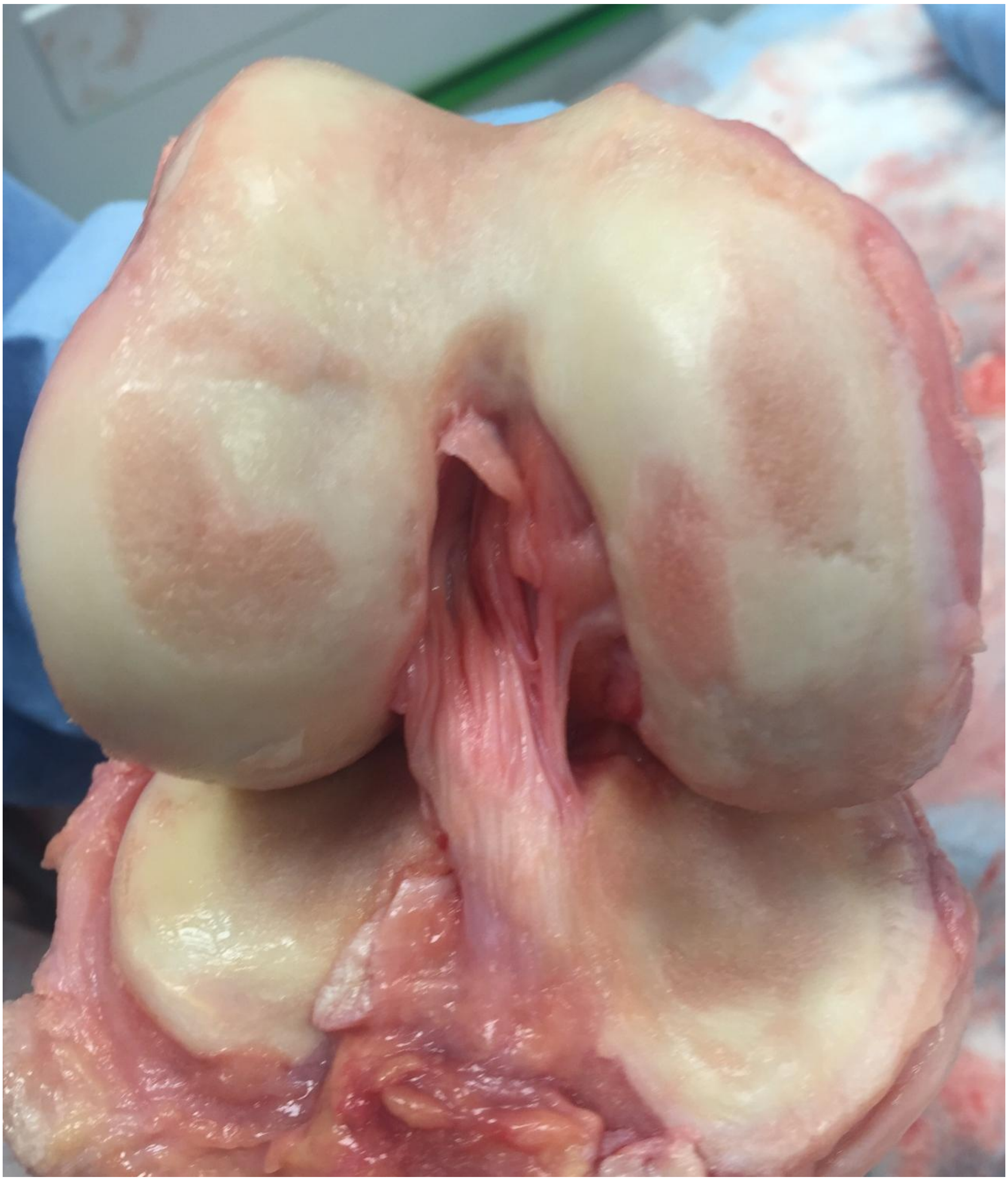

Figure S12. Cadaver 12, 88 years.
